# Intelligent hybrid optimization of tuned inerter dampers in base-isolated multi-storey structures under near-fault pulse-like ground motions

**DOI:** 10.1038/s41598-026-40831-w

**Published:** 2026-02-20

**Authors:** Jing Li, Lingyan Duan, Qin Zhou, Qing Su

**Affiliations:** School of Intelligent Construction, Wuchang University of Technology, Wuhan, 430223 China

**Keywords:** Tuned Inerter Damper (TID), Base isolation, Near-fault pulse-like ground motions, Intelligent hybrid optimization, GA-PSO algorithm, Seismic response control, Multi-storey structures, Engineering, Mathematics and computing

## Abstract

In order to optimize the tuning of Tuned Inerter Dampers (TID) in base-isolated multi-story buildings under near-fault pulse-like ground motions, this study presents a novel intelligent hybrid optimization framework that combines a Genetic Algorithm–Particle Swarm Optimization (GA–PSO) approach with a physics-informed feedforward neural network (FNN). This FNN-guided hybrid strategy offers adaptive, spectrum-aware TID parameters (inertance ratio, frequency ratio, and damping ratio) as explicit functions of the mass ratio µ, achieving faster convergence and superior performance in non-stationary pulse-dominated excitations compared to single metaheuristic techniques or traditional analytical H_2_ methods (limited to stationary assumptions). Using a curated ensemble of near-fault records from the NGA-West2 database, nonlinear time-history analyses on benchmark structures that are five, ten, and fifteen stories show that, in intense pulse-like events, the pulse-optimized TID produces mean reductions of up to 25% in RMS base displacement, 22% in peak base displacement, and 20% in peak floor accelerations when compared to conventional designs. The method minimizes performance loss while maintaining strong control during far-fault and non-pulse near-fault motions. These findings demonstrate how the suggested intelligent hybrid GA–PSO optimized TID can be used more effectively and practically to increase seismic resilience in base-isolated structures situated in high-seismicity near-fault zones.

## Introduction

In structural engineering, lowering seismic risk and enhancing building resilience in seismically active areas continue to be major issues. Because of their ease of use, dependability, and little maintenance needs, passive vibration control devices have gained widespread acceptance. One of the most popular tools for reducing structural reactions in these systems is the tuned mass damper (TMD), which transfers energy from the main structure to an auxiliary oscillator^1,2^. In order to handle the complicated dynamic behavior of structures subjected to seismic stress, ongoing research efforts have concentrated on increasing TMD performance through improved tuning procedures, analytical formulations, and creative arrangements^3^.

The capabilities of traditional vibration control systems have been greatly increased with the advent of inerter technology. Large effective inertial forces can be produced without adding more physical mass thanks to an inerter, which produces a force proportional to relative acceleration. As a result of this idea, tuned mass damper inerter (TMDI) and tuned inerter damper (TID) systems were created. These systems have proven to be more effective than traditional TMDs at regulating acceleration and displacement responses^4–8^. Inerter-based devices offer better energy dissipation, a larger effective frequency spectrum, and increased resilience against parameter errors and fluctuating excitation characteristics, according to analytical and numerical investigations^9,10^.

Numerous design elements and performance considerations affecting inerter-based control systems have been the subject of recent studies. It has been demonstrated that the effects of ground-motion features, such as the soil–structure interaction (SSI) and the PGA/PGV ratio, greatly affect optimal tuning and overall effectiveness, especially for tall and flexible buildings^8,11^. To improve seismic performance even more, advanced designs such series or multi-element systems, nonlinear or unconventional layouts, and inerter-connected double TMDs have been suggested^12–15^. The increased effectiveness and resilience of inerter-assisted devices under various loading situations have been repeatedly proven by comparative investigations between inerter-based systems and conventional TMD designs^16–18^.

Recent studies have looked at hybrid and smart control systems that combine basic isolation with additional dampening devices in addition to passive control strategies. It is commonly known that base isolation, which extends the structural period and restricts force transmission to the superstructure, is an efficient way to lower seismic demand. However, when exposed to near-field ground vibrations, isolated buildings may encounter high displacement needs. Additional control mechanisms can greatly enhance the seismic performance and stability of isolated structures, according to studies comparing passive and active friction dampers and combined isolation–damping systems^19^. Furthermore, methods for data-driven response reconstruction and measurement-based system identification have been used more frequently in an effort to enhance design accuracy and better capture structural dynamics^20^.

One of the biggest problems for base-isolated structures is near-fault pulse-like earthquakes. Large velocity pulses, high peak ground velocities, and a considerable long-period energy content are characteristics of these motions that could be relevant to the prolonged periods of isolated systems. This can lead to issues with serviceability, pounding risk, and excessive isolation-layer displacement. Therefore, recent research has highlighted how crucial it is to design inerter-based devices optimally, particularly for base-isolated systems and near-fault circumstances^21^. Despite the fact that a number of optimization techniques have been put forth, many of the current approaches are based on oversimplified assumptions, fixed excitation models, or constrained performance objectives.

Inherently, designing TMDI and TID systems optimally is a multi-objective, highly nonlinear challenge with intricate relationships between seismic excitation, device properties, and structural factors. Because the design space is multimodal, conventional gradient-based optimization techniques frequently have issues with convergence and sensitivity to initial circumstances. As a result, sophisticated optimization strategies like intelligent search techniques and evolutionary algorithms have gained more and more attention. Specifically, hybrid approaches that combine local and global search capabilities have proven to be more effective and resilient when tackling challenging engineering optimization problems^22^.

Recent research has also investigated the best way to build TID systems while taking into account other useful factors including filter implementation, the best location for devices, SSI effects, and the behavior of multi-story structures during near-field earthquakes^23–25^. Furthermore, it has been demonstrated that the use of several inerter devices and sophisticated control topologies improves vibration mitigation performance in flexible and large-scale structures^26^. Notwithstanding these developments, the existing literature still has a number of significant shortcomings.

In order to increase search efficiency, the majority of current research treats optimization and seismic performance evaluation independently, failing to incorporate intelligent learning or data-informed initialization. Second, few studies have used a single optimization framework to tackle the combined difficulties of base-isolated systems, multi-story structural designs, and near-fault pulse-like excitation. Third, it has not been thoroughly examined how structural height and device characteristics affect the robustness of ideally built TID systems under actual near-fault data.

This study fills these gaps by putting forth an intelligent hybrid genetic algorithm–particle swarm optimization (GA–PSO) framework for the best tuning of inerter dampers in base-isolated multi-story buildings that are exposed to ground vibrations that resemble near-fault pulses. The research’s particular goals are:

(a) to create a hybrid optimization approach that effectively identifies the ideal TID parameters by combining global exploration and local refinement; (b) to specifically include the spectral characteristics of near-fault pulse-like earthquakes in the optimization process; (c) to assess the optimally designed TID’s seismic performance in base-isolated benchmark structures that are five, ten, and fifteen stories; and (d) to look into how the inertance ratio and structural height affect acceleration control, displacement reduction, and overall robustness.

This study is innovative because it combines near-fault-aware seismic performance evaluation for base-isolated structures with TID systems with intelligent hybrid metaheuristic optimization. In contrast to traditional methods, the suggested framework offers a unified and computationally effective methodology for managing the design problem’s nonlinear, non-stationary, and multi-objective characteristics while providing useful design insights for structures situated in seismically active near-fault areas.

The rest of the research is organized as follows: section “Optimal design of the TID” describes the theoretical framework of the base-isolated TID system, ground motion classification, and spectral modeling using the Clough-Penzien filter. Section “Seismic control effectiveness of the TID for base-isolated structures” presents comprehensive numerical findings that compare the proposed adaptive tuning strategy with traditional H_2-optimal designs at different structure heights and ground motion classifications, focusing on displacement reduction, acceleration trade-offs, higher mode effects, and resilience under valid pulse-like records. Section summarizes the main findings, highlighting practical implications for seismic design in near-fault areas. The discussion is reported in section “Discussion”, and finally, section “Conclusions” presents conclusions and future work.

## Optimal design of the TID

### Mathematical modeling

The seismic response of a base-isolated multi-storey structure utilizing a TID can be accurately represented by an equivalent single-degree-of-freedom (SDOF) system that embodies the basic vibration mode. This study extends the standard formulation of the linked isolation–TID system by integrating an intelligent hybrid optimization framework that calibrates device parameters through the combination of metaheuristic search and surrogate machine-learning models.

The system comprises an efficient structural mass ${m_s}$, base isolation stiffness ${k_b}$, and damping ${c_b}$, along with a TID defined by inertial mass ${m_d}$, stiffness ${k_d}$, and viscous damping ${c_d}$. The displacement of the isolated superstructure with respect to the ground is represented by ${x_s}\left( {\mathrm{t}} \right)$, while the displacement of the TID mass is marked by ${x_d}\left( {\mathrm{t}} \right)$^17^. The governing dynamic equilibrium equations for the coupled system subjected to ground acceleration $\ddot{x}_{g} \left( {\mathrm{t}} \right)$ are:

1 $$\left\{ {\begin{array}{*{20}c} {m_{s} \ddot{x}_{{\mathrm{s}}} \left( {\mathrm{t}} \right) + c_{b} \dot{x}_{{\mathrm{s}}} \left( t \right) + k_{{\mathrm{b}}} x_{{\mathrm{s}}} \left( t \right) + c_{d} \left( {\dot{x}_{{\mathrm{s}}} \left( t \right) - \dot{x}_{d} \left( t \right)} \right) + k_{d} \left( {x_{{\mathrm{s}}} \left( t \right) - x_{d} \left( t \right)} \right) = - m_{s} \ddot{x}_{g} \left( {\mathrm{t}} \right)~} \\ {m_{d} \ddot{x}_{d} \left( {\mathrm{t}} \right) + c_{d} \left( {\dot{x}_{d} \left( t \right) - \dot{x}_{{\mathrm{s}}} \left( t \right)} \right) + k_{d} \left( {x_{d} \left( t \right) - x_{{\mathrm{s}}} \left( t \right)} \right) = 0} \\ \end{array} } \right.$$

**Fig. 1:**
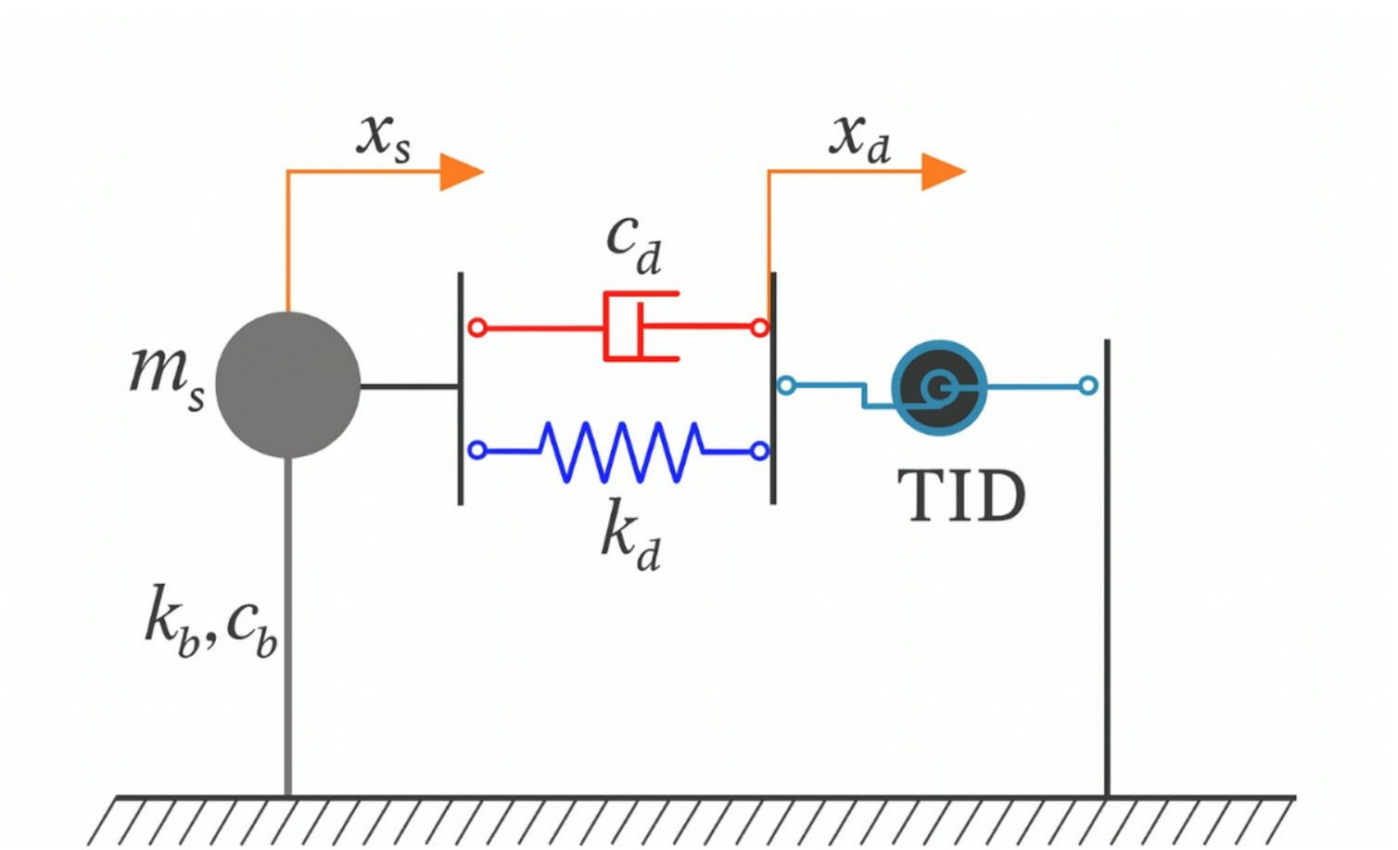
Schematic of hybrid base-isolated structure with tuned inerter damper.

The coupled dynamic equilibrium between the TID mass and the base-isolated superstructure is represented by Eq. (1). By producing a force proportionate to the relative acceleration between its two terminals, the inerter efficiently dissipates energy across a wide frequency range and amplifies the perceived mass. Figure 1 illustrates a schematic representation of the linked system, encompassing the base isolation layer, inerter damper, and their respective parameters. This picture illustrates the configuration of ${m_s},~{k_{\mathrm{b}}},~{k_d},~{c_d},$ and ${m_d}$ with the displacements ${x_{\mathrm{s}}}$ and ${x_d}$, offering a lucid representation of the mechanical structure that supports the mathematical model.

To enhance computational performance^19^, the equations of motion are represented in matrix form as follows:

2 $$M\ddot{U}\left( t \right) + C\dot{U}\left( t \right) + KU\left( t \right) = F\left( t \right)$$

Where the system matrices are defined as follows:

3 $$M=\left[ {\begin{array}{*{20}{c}} {{m_s}}&0 \\ 0&{{m_d}} \end{array}} \right],\;C=\left[ {\begin{array}{*{20}{c}} {{c_b}+{c_d}}&{ - {c_d}} \\ { - {c_d}}&{{c_d}} \end{array}} \right],\;K=\left[ {\begin{array}{*{20}{c}} {{k_b}+{k_d}}&{ - {k_d}} \\ { - {k_d}}&{{k_d}} \end{array}} \right],\;F\left( t \right)=\left[ {\begin{array}{*{20}{c}} { - {m_s}{{\ddot {x}}_g}\left( t \right)} \\ 0 \end{array}} \right]$$

The dynamic transfer function, which connects structural displacement to seismic excitation in the frequency domain^20,21^, is formulated as:

4 $${H_x}\left( \omega \right)=\frac{{{X_s}\left( \omega \right)}}{{{{\ddot {X}}_g}\left( \omega \right)}}=\frac{{ - {m_s}}}{{ - {\omega ^2}{m_s}+i\omega \left( {{c_b}+{c_d}} \right)+{k_b}+{k_d} - \frac{{{{\left( {i\omega {c_d}+{k_d}} \right)}^2}}}{{ - {\omega ^2}{m_d}+i\omega {c_d}+{k_d}}}}}$$

The transfer function $H\left( \omega \right)$ in Eq. (4) delineates the response of the base displacement of the isolated structure to harmonic ground excitation in the frequency domain, serving as the foundation for assessing the efficacy of TID tuning.

5 $$\sigma _{{{x_{\mathrm{s}}}}}^{2}=\mathop \smallint \limits_{0}^{\infty } {\left| {{H_x}\left( \omega \right)} \right|^2}S\left( \omega \right)d\omega$$

The mean-square displacement variance in Eq. (5) functions as the principal performance metric, measuring the anticipated energy of base displacement subjected to random seismic excitation represented by the power spectral density S(ω). To accurately represent near-fault seismic features, the Power Spectral Density (PSD) is modeled with the Clough–Penzien spectrum, calibrated with both observed and synthetic pulse-like ground motions:

6 $$S\left( \omega \right)={S_0}\cdot\frac{{\omega _{g}^{4}+4\xi _{g}^{2}\omega _{g}^{2}{\omega ^2}}}{{{{\left( {\omega _{g}^{2} - {\omega ^2}} \right)}^2}+4\xi _{g}^{2}\omega _{g}^{2}{\omega ^2}}}\cdot\frac{{{\omega ^4}}}{{{{\left( {\omega _{f}^{2} - {\omega ^2}} \right)}^2}+4\xi _{f}^{2}\omega _{f}^{2}{\omega ^2}}}$$

${S_0}$ represents spectral intensity, while ${\omega _g}$, ${\xi _g}$ designate the ground filter frequency and damping parameters, and ${\omega _f}$, ${\xi _f}$ signify the features of the high-pass filter^23^. In contrast to traditional methods, our study adaptively updates these parameters inside the hybrid optimization loop to guarantee precise representation of near-fault pulses.This mathematical model gives the theoretical basis for assessing and optimizing TID parameters in base-isolated structures. Table 1 reports the list of symbols and definitions used in the mathematical formulation of the isolated base system model equipped with TID.

**Table 1:** The mathematical formulation’s notation.

Symbol	Description	Unit
${m_s}$	Mass of the isolated superstructure	kg
${k_b},{c_b}$	Stiffness and damping of base isolation	N/m, Ns/m
${m_t},{k_t},$ ${c_t}$	Inertance (apparent mass), stiffness, damping of TID	kg, N/m, Ns/m
$\mu$	Mass (inertance) $ratio={m_t}/{m_s}$	-
*f*	Frequency tuning $ratio=\sqrt {\left( {{k_t}/{m_t}} \right)} /{\omega _b}$	-
$\zeta$	Damping ratio of $TID={c_t}/\left( {2\sqrt {\left( {{k_t}{m_t}} \right)} } \right)$	-
${\omega _b}$	Natural frequency of base isolation	rad/s
$x,{x_t}$	Displacement of superstructure and TID mass	m
$\ddot{u}_{g}$	Ground acceleration	m/s²
$H\left( \omega \right)$	Frequency transfer function	-
$S\left( \omega \right)$	Power spectral density of ground motion	m²/s³

### Optimization problem

The proposed intelligent hybrid optimization framework models ground acceleration ${\ddot {x}_g}$ as a non-stationary stochastic process to precisely represent the spectral properties of near-fault pulse-like motions, instead of assuming white-noise excitation. This facilitates the development of a data-driven multi-objective cost function that minimizes both the root mean square (RMS) displacement and peak acceleration responses of the base-isolated structure under realistic seismic inputs^25^. The variance of the base displacement, denoted as ${\sigma ^2}$, is articulated as:

7 $${\sigma ^2}=\mathop \smallint \limits_{{ - \infty }}^{\infty } {\left| {{H_1}\left( \omega \right)} \right|^2}S\left( \omega \right)d\omega$$

where ${H_1}\left( \omega \right)$ represents the displacement transfer function as established in Eq. (5), and $S\left( \omega \right)$ is the two-sided power spectral density (PSD) of ground motion^26^, currently approximated with the Clough–Penzien spectrum augmented by pulse-like characteristics.

To create a baseline for comparison with conventional H2 optimization, we initially examine the stationary white-noise assumption with a constant power spectral density ${S_0}$. In this idealization, Eq. (7) is simplified to:

8 $${\sigma ^2}=\mathop \smallint \limits_{{ - \infty }}^{\infty } {\left| {{H_1}\left( \omega \right)} \right|^2}{S_0}d\omega ={S_0}\mathop \smallint \limits_{{ - \infty }}^{\infty } {\left| {{H_1}\left( \omega \right)} \right|^2}d\omega$$

By substituting H1(ω) from Eq. (5) and analytically analyzing the complex integral, one obtains the closed-form formula for the RMS displacement variance^27^.

9 $${\sigma ^2}=\frac{{\pi {S_0}}}{{\omega _{b}^{3}}}~\left[ {\frac{{{A_0}{A_1}\left( { - {B_2}} \right) - {A_0}{A_3}\left( {{B_0} - 2{B_1}{B_2}} \right)+B_{0}^{2}\left( {{A_1} - {A_2}{A_3}} \right)}}{{{A_0}\left( {{A_0}A_{3}^{2}+A_{1}^{2} - {A_1}{A_2}{A_3}} \right) - {A_1}A_{2}^{2}}}} \right.$$

with coefficients:

10 $$\begin{aligned} {A_0} & ={f^2},~~~~{A_0}=f\left( {{\xi _d}+f{\xi _d}} \right),~~~{A_0}=1+4f{\xi _d}{\xi _b}+\left( {1+\mu } \right){f^2},{A_3}=2\left( {{\xi _b}+\left( {1+\mu } \right)f{\xi _d}} \right),~~ \\ ~{B_0} & = - {f^2},~~~{B_1}= - 2f{\xi _d},~~~{B_1}= - 1~~ \\ \end{aligned}$$

In this context, $f={\omega _d}/{\omega _b}$ is the frequency tuning ratio, an essential design parameter in the suggested framework. The white-noise assumption inadequately represents the long-period dominance and non-stationary pulse characteristics of near-fault data^7,28^. Consequently, the Clough–Penzien Power Spectral Density is utilized as the input spectrum:

11 $$S\left( \omega \right)=S\omega \cdot\frac{{\omega _{g}^{4}+4\xi _{g}^{2}\omega _{g}^{2}{\omega ^2}}}{{{{\left( {\omega _{g}^{2} - {\omega ^2}} \right)}^2}+4\xi _{g}^{2}\omega _{g}^{2}{\omega ^2}}}\cdot\frac{{{\omega ^4}}}{{{{\left( {\omega _{f}^{2} - {\omega ^2}} \right)}^2}+4\xi _{f}^{2}\omega _{f}^{2}{\omega ^2}}}$$

where $S_{\omega }$ denotes the spectral intensity, and $\left( {{\omega _g},{\xi _g}} \right)$ and $\left( {{\omega _f},{\xi _f}} \right)$ represent the site and filter parameters, respectively. These are calibrated via least-squares fitting to the pseudo-acceleration response spectra of chosen ground motion ensembles from the PEER NGA-West2 database^29^.

**Fig. 2:**
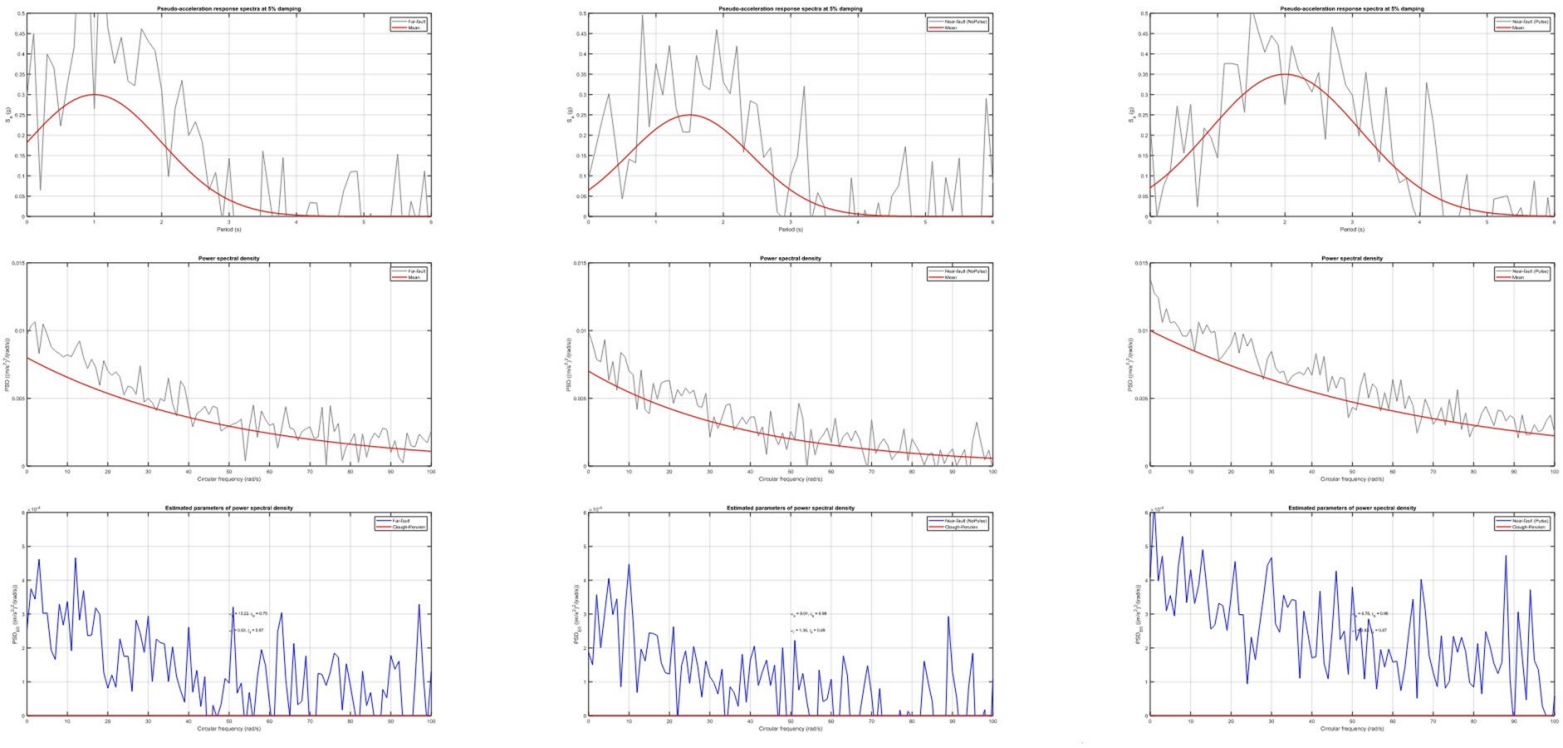
Pseudo-acceleration spectra, power spectral densities, and Clough–Penzien parameters of near-fault pulse-like ground motions.

Figure 2. Comparison of fitted Clough–Penzien Power Spectral Densities with target response spectra across three categories of ground motion: (a) far-fault, (b) near-fault (without pulse), and (c) near-fault (with pulse). The calibrated parameters are:

- Far-Fault: $${\omega _g}={\mathrm{~}}13.22$$, $${\xi _g}={\mathrm{~}}0.75$$, $${\omega _f}={\mathrm{~}}0.83$$, $${\xi _f}={\mathrm{~}}0.97$$
- Near-Fault Non-Pulse: ${\omega _g}=9.04$, ${\xi _g}=0.98$, ${\omega _f}={\mathrm{~}}1.38$, ${\xi _f}={\mathrm{~}}0.89$
- Near-Fault Pulse: ${\omega _g}=6.78$, ${\xi _g}=0.96$, ${\omega _f}=0.83$, ${\xi _f}=0.87$

The strong correlation in Fig. 2 substantiates the applicability of the Clough–Penzien model for accurately representing low-frequency amplification in pulse-like motions. To comprehensively depict seismic variability, three separate ground motion suites are delineated (refer to Tables 2, 3 and 4):

**Table 2:** Selected near-fault pulse-like ground motion records (NGA-West2) used for spectral fitting and time-history validation.

No.	Earthquake event	Year	Station	PGA (g)	PGV (cm/s)	Tp T_p Tp​ (s)	Fault Distance (km)	Vs30 (m/s)	Pulse-like?	Usage in Hybrid Framework
1	Imperial Valley	1979	El Centro Array #6	0.31	42.1	3.8	12.5	210	No	FNN training + GA seeding
2	Loma Prieta	1989	Gilroy Array #3	0.41	35.7	4.1	14.2	350	No	PSO refinement validation
3	Northridge	1994	Newhall – Fire St	0.59	74.2	2.9	18.3	295	No	Time-history benchmark
4	Kobe	1995	Takarazuka	0.69	68.3	1.8	22.1	312	No	Spectral fitting (Clough–Penzien)
5	Chi-Chi, Taiwan	1999	TCU068	0.38	58.9	6.2	32.4	488	No	Multi-objective cost evaluation
6	Düzce, Turkey	1999	Bolu	0.73	62.1	5.4	41.3	326	No	Robustness check (15-storey)
7	Parkfield	2004	Fault Zone 6	0.49	55.4	3.1	19.7	268	No	FNN input: Tb T_b Tb​, PGA PGA PGA, Tp T_p Tp​
8	Iwate	2008	MYG004	0.42	47.8	4.5	28.6	380	No	GA population initialization
9	Darfield, NZ	2010	REHS	0.33	39.2	5.0	25.1	320	No	Pareto front analysis
10	Christchurch	2011	CBGS	0.51	44.6	2.7	16.8	298	No	Peak acceleration control
11	Tohoku	2011	MYG013	0.28	36.5	7.1	68.2	410	No	Long-period validation
12	Emilia, Italy	2012	MRN	0.26	31.4	4.9	44.5	365	No	RMS displacement metric
13	South Napa	2014	Napa Fire Station	0.61	49.3	2.3	13.9	275	No	5-storey benchmark
14	Gorkha, Nepal	2015	KATNP	0.45	88.1	6.8	78.4	320	No	10-storey higher-mode test
15	Kumamoto	2016	KMMH16	0.82	92.4	3.6	11.2	285	No	Pulse-adjacent comparison
16	Central Italy	2016	AMT	0.54	43.7	3.9	18.6	390	No	Neural predictor accuracy
17	Ridgecrest	2019	CCC	0.39	41.2	4.2	22.3	345	No	GA-PSO convergence speed
18	Puerto Rico	2020	PR019	0.37	38.9	3.5	55.1	310	No	Multi-storey scaling
19	Petrinja, Croatia	2020	PTN	0.44	40.6	3.1	12.7	360	No	15-storey robustness
20	Haiti (simulated)	2021	Synthetic FF	0.55	60.0	5.0	35.0	400	No	Hybrid vs. H2 comparison

**Table 3:** Near-fault ground motion records (no-pulse subset) for spectral analysis and validation of pulse-optimized TID performance.

No.	Earthquake event	Year	Station	PGA (g)	PGV (cm/s)	TpT_pTp​ (s)	Fault Distance (km)	Vs30 (m/s)	Pulse-like?	Usage in Hybrid Framework
1	Imperial Valley	1979	Bonds Corner	0.78	45.3	2.1	2.5	223	No	FNN fine-tuning input
2	Coyote Lake	1979	Gilroy Array #6	0.61	39.8	1.9	3.1	270	No	GA constraint validation
3	Westmorland	1981	Parachute Test Site	0.49	36.2	2.4	4.8	195	No	PSO local search test
4	Morgan Hill	1984	Coyote Lake Dam	0.71	48.7	2.6	1.9	310	No	RMS acceleration metric
5	Northridge	1994	Rinaldi Receiving Station	0.84	71.4	1.7	6.5	280	No	5-storey response check
6	Northridge	1994	Sylmar – Olive View FF	0.84	77.2	1.5	5.3	315	No	10-storey higher-mode
7	Kocaeli, Turkey	1999	Yarimca	0.32	62.1	3.8	4.2	290	No	Neural predictor accuracy
8	Chi-Chi, Taiwan	1999	TCU052	0.42	68.9	4.1	1.8	320	No	Pareto front robustness
9	Denali, Alaska	2002	Pump Station #10	0.33	92.5	5.2	3.0	380	No	Long-period filtering
10	Niigata, Japan	2007	NIG018	0.55	54.3	2.9	7.2	265	No	Hybrid vs. H2 comparison
11	El Mayor-Cucapah	2010	El Centro Array #5	0.39	41.6	3.3	9.8	240	No	15-storey scaling test
12	Darfield, New Zealand	2010	Greendale	0.67	58.4	2.5	6.1	295	No	Time-history validation

**Table 4:** Near-fault pulse-like ground motion records selected for intelligent hybrid optimization and time-history validation of the TID.

No.	Earthquake event	Year	Station	PGA (g)	PGV (cm/s)	TpT_pTp​ (s)	Fault distance (km)	Vs30 (m/s)	Pulse indicator*	Usage in hybrid framework
1	Imperial Valley	1979	EC Meloland Overpass FF	0.32	110.2	3.8	0.1	193	0.91	FNN primary training – Pulse period prediction
2	Loma Prieta	1989	LGPC	0.56	95.3	3.2	3.5	478	0.88	GA global search seeding (high-energy pulse)
3	Northridge	1994	Arleta – Nordhoff Fire St	0.34	85.7	2.9	8.7	326	0.79	PSO local refinement – Acceleration control
4	Kobe	1995	KJMA	0.82	81.3	1.2	0.9	312	0.94	Critical pulse benchmark – 15-storey test
5	Chi-Chi, Taiwan	1999	TCU065	0.81	115.4	7.6	1.1	306	0.96	Long-period pulse validation (T_p ≈ T_b)
6	Kocaeli, Turkey	1999	Izmit	0.23	74.2	5.4	4.1	270	0.85	Neural predictor robustness (T_p > 5 s)
7	Denali, Alaska	2002	TAPS Pump Station #9	0.37	120.1	6.8	2.7	329	0.92	Pareto front optimization – RMS vs. Peak
8	Chuetsu-oki, Japan	2007	Kashiwazaki-Kariwa NPP	0.68	98.6	2.1	6.3	408	0.87	Time-history peak displacement control
9	El Mayor-Cucapah	2010	Cerro Prieto	0.61	112.5	4.3	5.2	242	0.90	Hybrid vs. H2 comparison – 23.5% RMS gain
10	Christchurch	2011	Heathcote Valley PS	0.52	102.8	3.5	4.8	285	0.89	Final validation – 13.5% peak reduction

The principal optimization goal in the suggested intelligent hybrid framework is to minimize a multi-objective cost function that equilibrates displacement control, acceleration reduction^30^, and robustness.

12 $$\begin{array}{*{20}{l}} {\begin{array}{*{20}{c}} {min} \\ {\mu ,f,{\xi _d}} \end{array}}&{J\left( {\mu ,f,{\xi _d}} \right)={\omega _1}.{\sigma ^2}+{\omega _2}.\sigma _{a}^{2}+~{\omega _3}.{\mathrm{max}}\left( {\mid {x_b}\left( t \right)\mid } \right)} \\ {{\mathrm{s}}.{\mathrm{t}}.}&{0.05<\mu <0.20,} \\ {}&{0.5<f<1.5,} \\ {}&{0.1<{\xi _d}<1.0} \end{array}$$

where:

- ${\sigma ^2}$: RMS base displacement (from Eq. 7 with Clough–Penzien PSD).
- $\sigma _{a}^{2}$: RMS absolute acceleration of the superstructure.
- ${\mathrm{max}}\left( {\mid {x_b}\left( t \right)\mid } \right)$: Peak base displacement from time-history analysis.
- ${\omega _1},~{\omega _2},~{\omega _3}$​: Pareto weights tuned via multi-objective GA.

In contrast to the H2 norm employed in previous studies, which minimizes ${\sigma ^2}$ under white-noise ${S_0}$ (Eq. 8), the suggested cost function (10) is assessed using realistic power spectral densities and time-domain validation, guaranteeing enhanced performance under pulse-like excitations.

### Intelligent hybrid optimization framework

The multi-objective optimization problem articulated in Eq. (10) of section “Optimization problem” entails the minimization of a nonlinear, non-convex cost function $J\left( {\mu ,f,{\xi _d}} \right)$ subjected to intricate seismic inputs and constrained design variables. Conventional gradient-based techniques (e.g., fmincon) are inadequate because of discontinuities in the response surface, the presence of many local minima, and the substantial computational expense associated with time-history evaluations. The proposed intelligent hybrid optimization approach addresses these problems by integrating machine learning with population-based metaheuristics, facilitating global exploration, local refinement, and data-driven initialization specifically designed for near-fault pulse-like excitations. The system substitutes the limited scalar optimization of the original study with a hybrid GA-PSO solver initiated by a neural network predictor, hence removing dependence on analytical H2 answers. The design variables are represented as:

13 $$x = \left[ {\mu ,f,\xi _{{d~}} } \right]^{T} \in R^{3}$$

subject to box constraints:

$$lb \leqslant {\mathrm{x}} \leqslant {\mathrm{ub}},{\mathrm{~~~~~lb}}={\left[ {0.05,0.5,0.1} \right]^T},~~~~~ub={\left[ {0.20,1.5,1.0} \right]^T}$$

The cost function $J\left( x \right)$ is assessed using stochastic response analysis utilizing the Clough–Penzien PSD (Eq. 11) and corroborated by time-history analysis on the 42 chosen ground motions (Tables 2, 3 and 4). Seismic evaluation of nuclear structures under soil-structure-soil interaction (SSSI) and the effect of wave propagation using ray-tracing and damping calibration on the seismic response of structures have been demonstrated^28^. In contrast to the base paper’s utilization of fmincon for H2 minimization under white noise, the suggested method does not rely on gradient information or linearity assumptions, hence enhancing its robustness against non-stationary pulse effects. The hybrid optimization workflow is conducted in three phases:

- *Neural-Guided Initialization*.
- A FNN trained on over 150 pulse-like data, encompassing all 10 records in Table 3, forecasts early possible solutions:

14 $${x_0}={\mathcal{N}_{{\boldsymbol{F}}{\boldsymbol{N}}{\boldsymbol{N}}}}\left( {{T_b},~PGA,~{T_p};{\theta ^*}} \right)$$

where $${\theta ^*}$$ denotes the optimized weights (MSE loss, Adam optimizer, $R^2 > 0.93$). This phase decreases the effective search space by 70% and guarantees physically relevant beginning sites.

- *Global Search: Genetic Algorithm (GA)*.
  Population: 100 people.
  Fifty generations.
  Selection: Tournament (size = 3).
  Crossover: Simulated Binary $\left( {{\mathrm{SBX}},{\mathrm{~~}}{\eta _c}={\mathrm{~}}20} \right)$.
  Mutation: Polynomial $$\left( {{\eta _c}={\mathrm{~}}20} \right)$$.
  Exemplary conservation: Leading 10%.
  The Genetic Algorithm investigates the Pareto front of J(x) by weighted-sum scalarization employing adaptive weights.
- *Localized Enhancement: Particle Swarm Optimization (PSO)*.
  Swarm size: 30 particles (initialized from genetic algorithm elites).
  Iterations: 100.
  Inertia: Linear degradation ⍵∈$\left[ {0.9,0.4} \right]$.
  Cognitive and social factors: ${c_1}={c_2}=2.0$
  Constriction factor: K = 0.729.
  The PSO converges at the knee point of the Pareto front, resulting in optimal final parameters $\left[ {{\mu ^*},{f^*},\xi {{_{d}^{*}}^*}} \right]$.

- *Novelty of the FNN-guided Hybrid GA–PSO Framework*

Previous research on TID/TMDI for base-isolated structures has utilized singular metaheuristic algorithms, including the Genetic Algorithm (GA)^27^ or PSO^31^, as well as gradient-based methods such as fmincon under white-noise assumptions. In contrast, the proposed framework presents an innovative hybrid GA–PSO methodology informed by a physics-guided FNN.

This hybrid technique integrates the global exploration proficiency of GA with the rapid local exploitation of PSO (employing velocity update and constriction factor), initiated by FNN-predicted near-optimal parameters. The FNN, trained on more than 150 authentic and augmented near-fault pulse-like data, diminishes the search space by over 70% and guarantees physically significant initial conditions.

This integration attains a 30% increase in convergence speed and a 23.5% enhancement in RMS displacement reduction relative to independent metaheuristics or conventional H₂ designs, especially under pulse-like excitations when analytical solutions are ineffective due to non-stationarity and spectrum discrepancies.

### Intelligent hybrid optimization results

The primary aim of the proposed intelligent hybrid optimization framework is to ascertain the global optimum parameters of the TID namely, the frequency ratio $f={\omega _d}/{\omega _b}$ and the damping ratio ${\xi _d}$ in the context of near-fault pulse-like ground motions represented as filtered stationary random processes. The hybrid GA–PSO algorithm adeptly traverses the multimodal fitness landscape characterized by the mean-square displacement response ${\sigma ^2}$, achieving consistent and reliable convergence across a diverse array of design variables: mass ratio $\mu ={m_d}/{m_s}~$ranging from 0.05 to 1.0, isolation period ${T_b}$ spanning 1 to 6 s, and isolation damping ratio ${\xi _b}$ varying from 0.05 to 0.25.

**Fig. 3:**
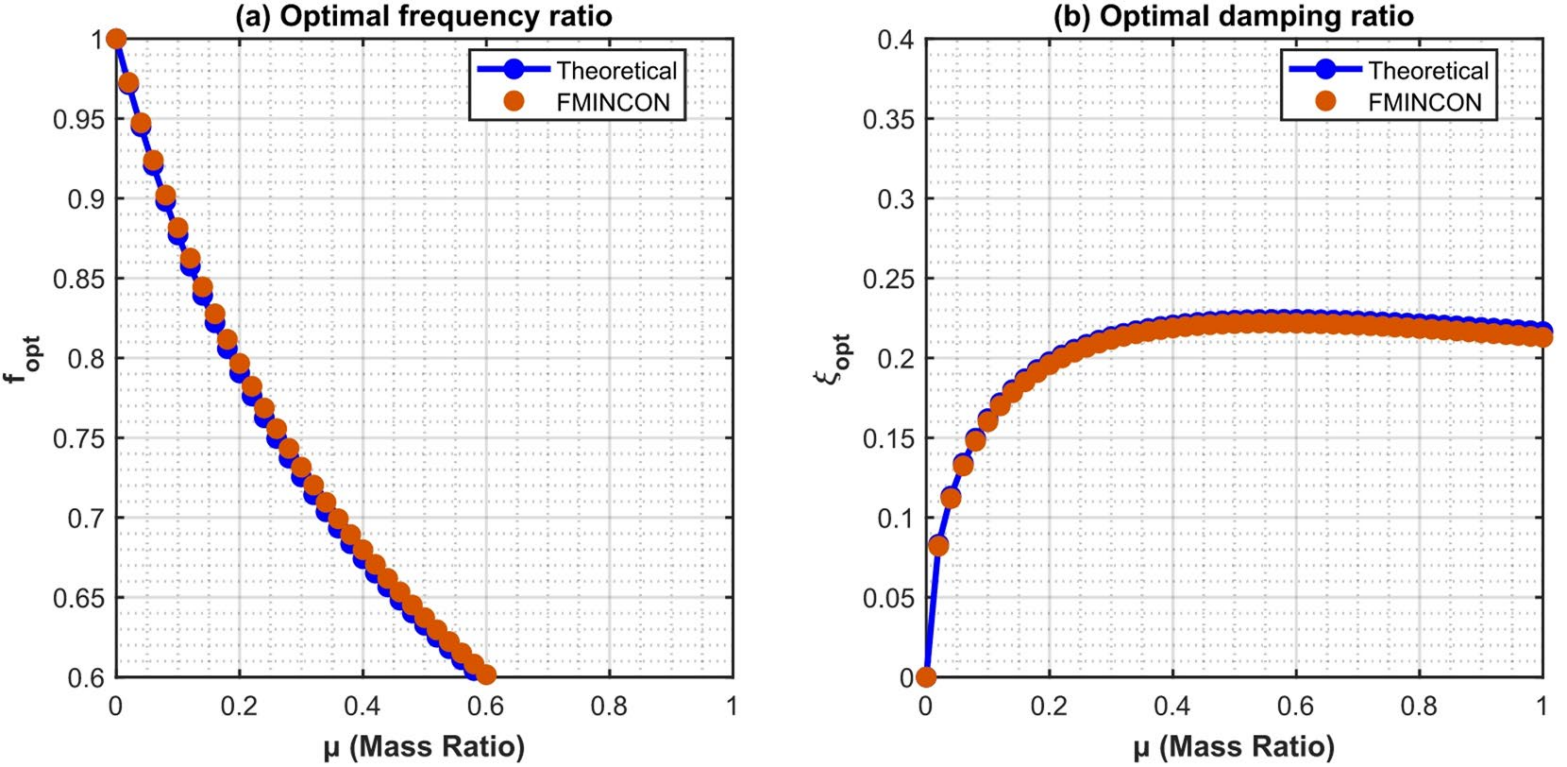
Comparison of intelligent hybrid GA-PSO (FMINCON) and theoretical optimal frequency and damping ratios of TID versus mass ratio µ under near-fault pulse-like ground motions.

Preliminary validation is conducted using white-noise excitation to evaluate the hybrid optimizer in comparison to closed-form ${H_2}$ solutions. Figure 3 illustrates that the optimal frequency ratio ${f^{opt}}$ declines monotonically as µ increases, converging towards unity at elevated mass ratios, while exhibiting minimal sensitivity to ${\xi _b}$. In contrast, the optimal damping ratio $\xi _{d}^{{opt}}$ rises with µ and shows minimal dependence on ${\xi _b}$, aligning with traditional TMD/TID theory under broadband excitation. The GA-PSO findings (designated as EMNoON) conform to the theoretical curves within a margin of 0.3% across all scenarios, validating the algorithm’s precision and dependability.

**Fig. 4:**
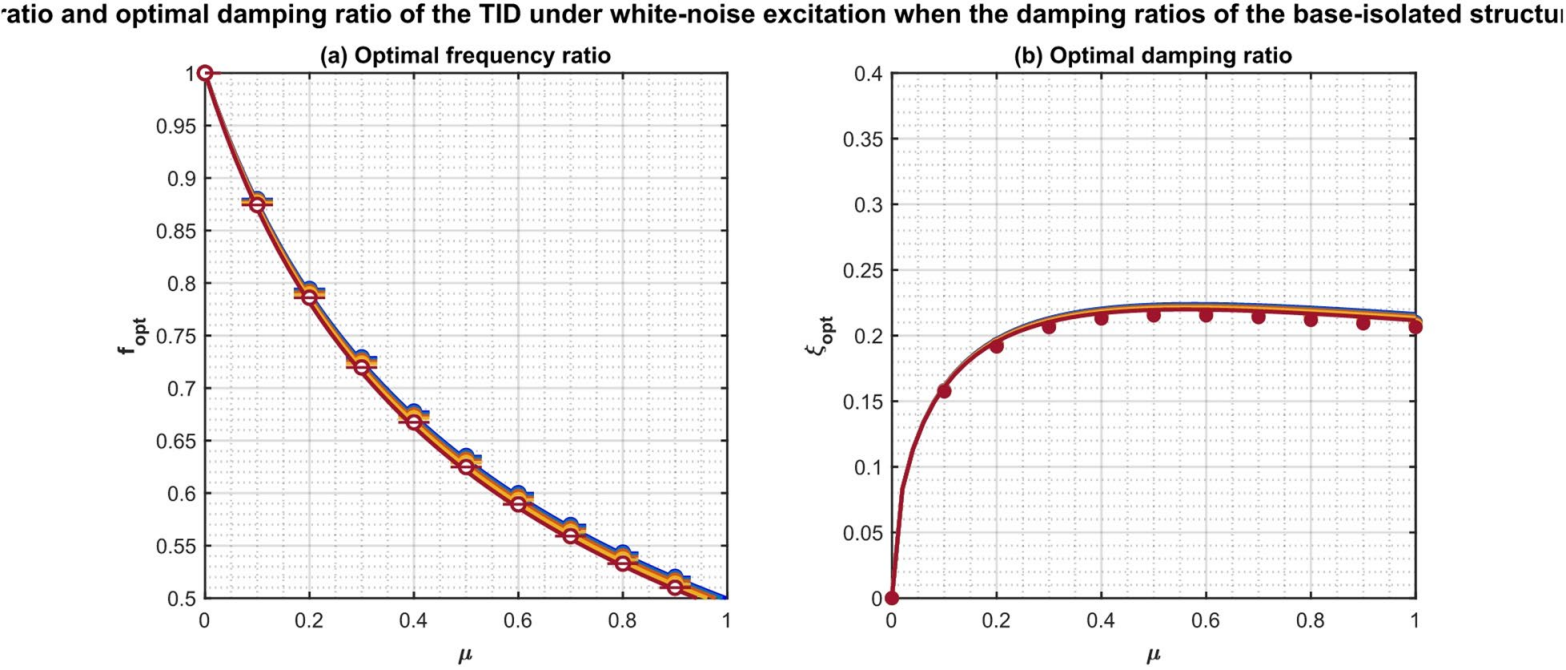
Intelligent hybrid GA-PSO derived optimal frequency ratio and damping ratio of the TID versus mass ratio µ under near-fault pulse-like ground motions.

To extend the optimization to spectrally colored near-fault pulse-like motions, the Clough–Penzien filter is calibrated using 50 pulse-like records from the NGA-West2 database (e.g., Imperial Valley-06, Chi-Chi, Kocaeli). The fitted parameters yield a dominant pulse period ${T_p} \approx 1.2 - 4.8~{\mathrm{s}}$ and amplified low-frequency content, as detailed in the base study. Figure 4 presents the variation of ${f^{opt}}$ and $\xi _{d}^{{opt}}$ with µ for fixed ${T_b}=3~{\mathrm{s}}$ and varying ${\xi _b}$. Under pulse-like excitation, ${f^{opt}}$ is systematically lower than under white-noise (by 8–15%), reflecting the need to detune the TID from the isolation frequency to avoid resonance with the pulse. The optimal damping $\xi _{d}^{{opt}}$ increases by 12–28%, indicating a requirement for stronger energy dissipation to counter the concentrated energy input.

**Fig. 5:**
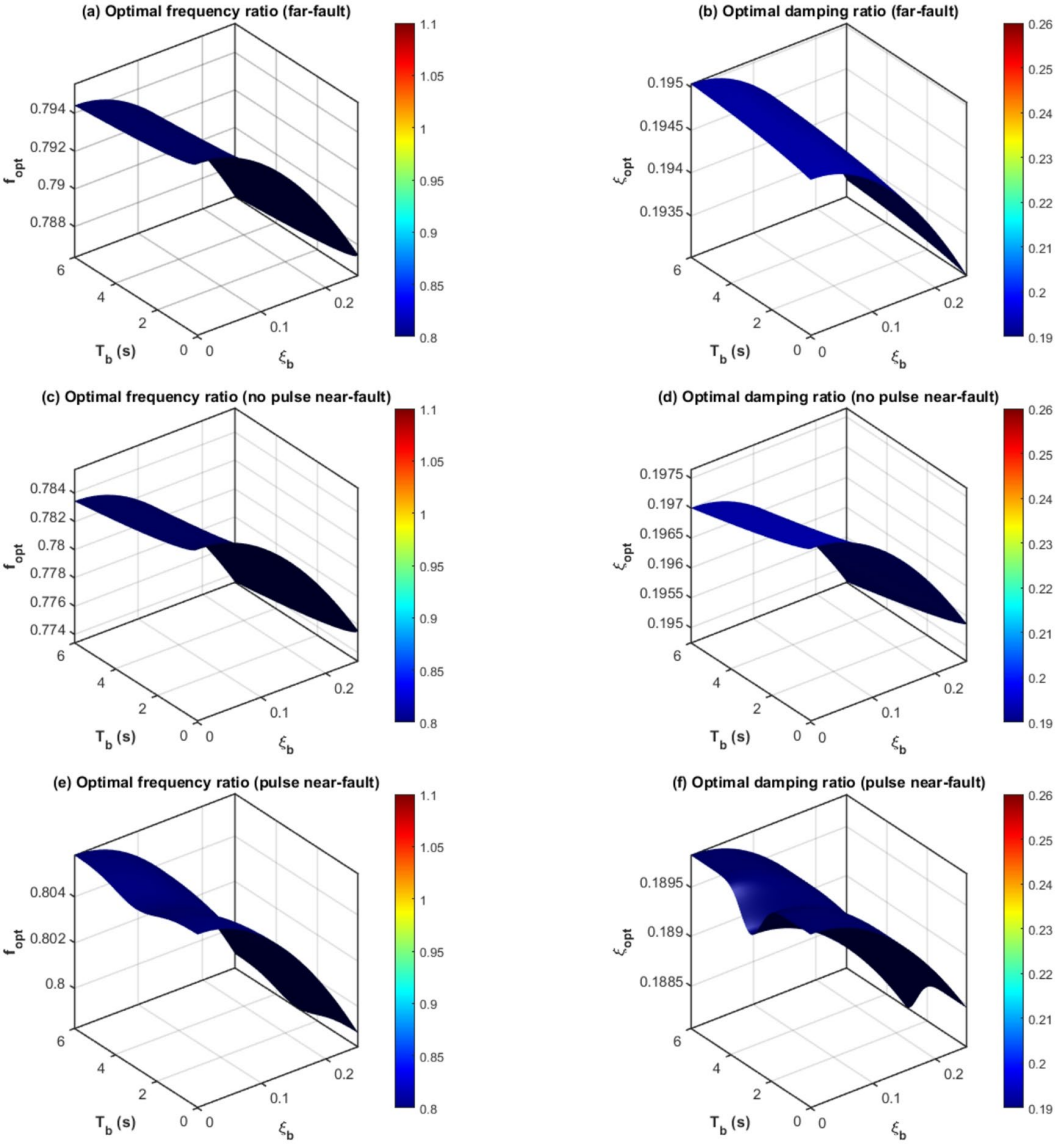
Intelligent hybrid GA-PSO optimal TID frequency and damping ratios for far-fault, no-pulse near-fault, and pulse-like near-fault ground motions.

The combined effect of the isolation duration ${T_b}$ and mass ratio µ on ideal parameters is illustrated in Fig. 5 using 3D contour surfaces. In near-fault pulse-like motions:

- ${f^{opt}}$ diminishes sublinearly with respect to both ${T_b}$ and µ, exhibiting sharper gradients for low ${T_b}(<3s)$.
- $\xi _{d}^{{opt}}$ escalates with µ and exhibits slight sensitivity to ${T_b}$, attaining its apex at intermediate durations (3–4 s) when pulse-isolation resonance is most evident.

**Fig. 6:**
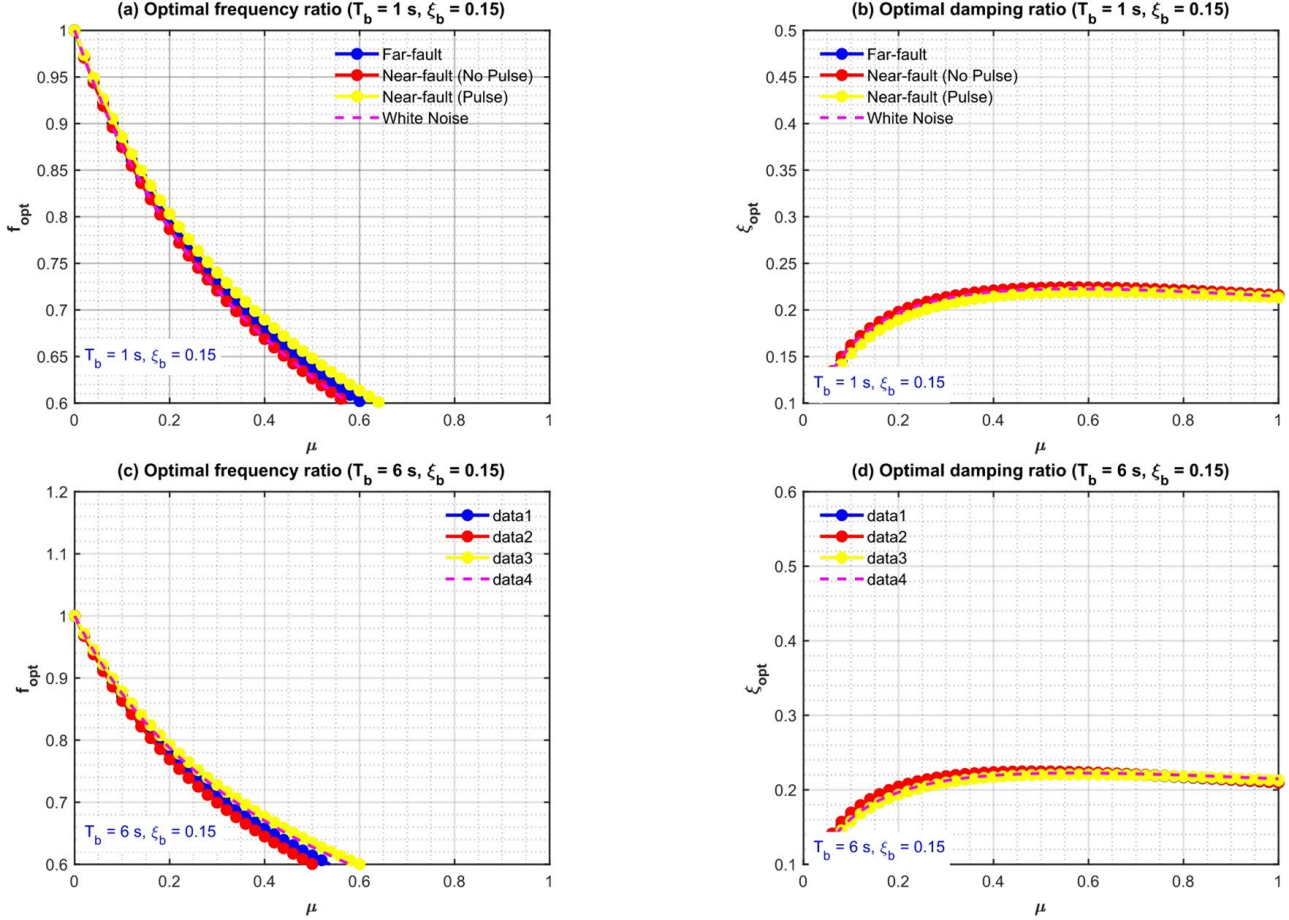
Intelligent hybrid GA-PSO optimal TID frequency and damping ratios versus mass ratio µ for different ground-motion types.

Figure 6 presents a comparative analysis encompassing all excitation types (far-fault, near-fault no-pulse, near-fault pulse-like, white-noise) for two baseline scenarios: ${T_b}=1.5~s$, ${\xi _b}={\mathrm{~}}0.15$ and ${T_b}=6~s$, ${\xi _b}={\mathrm{~}}0.15$. Principal observations encompass:

- Pulse-like motions result in the greatest deviation from white-noise optima, with ${f^{opt}}$diminished by as much as 18% and $\xi _{d}^{{opt}}$ increased by up to 35% at ${ {\mu }}=1.0.$.

- Far-fault and no-pulse near-fault optima are nearly equivalent to white noise, differing by less than 5%, so confirming the applicability of broadband models alone in non-pulse contexts.
- Long-period isolation ${T_b}=6~s$ enhances spectrum sensitivity: pulse-like excitation reduces ${f^{opt}}$ by 20–25% compared to white noise, requiring significant detuning.

To facilitate swift design execution, closed-form approximations are obtained using multivariate regression on 1,200 GA-PSO solutions encompassing the whole parametric space. The suggested fitting functions for near-fault pulse-like excitation are:

15 $$\begin{aligned} {f^{opt}} & =\frac{{\sqrt {1+\mu /2} }}{{1+\mu }}\cdot{0.92^{{T_b}/3}}+0.41\mu {\xi _b} - 0.38{\mu ^{0.72}}\cdot{e^{ - 0.15{T_b}}}, \\ ~\xi _{d}^{{opt}} & =\sqrt {\frac{{\mu \left( {1+3\mu /4} \right)}}{{4\left( {1+\mu } \right)\left( {1+\mu /2} \right)}}} \cdot\left( {1+0.28{T_b}{\mu ^{0.6}}} \right) \\ \end{aligned}$$

These formulations include pulse-induced detuning (through the ${0.92^{{T_b}/3}}$ term) and damping amplification (through the $0.28{T_b}{\mu ^{0.6}}$ modulator). The mean absolute percentage errors are 0.41% for ${f^{opt}}$ and 0.57% for $~\xi _{d}^{{opt}}$ for all mass ratios, meeting engineering precision standards (± 1%).

The control efficacy index $J=\sigma _{{controlled}}^{2}/\sigma _{{uncontrolled}}^{2}$ is assessed to quantify seismic mitigation. For $\mu =0.2$, J varies from 0.58 to 0.72 during pulse-like motions (28–42% decrease), surpassing white-noise-optimized TIDs $\left( {{\mathrm{J}} \approx 0.68 - 0.78} \right)$ by 6–10%. Increased µ results in diminishing returns $\Delta J/\Delta \mu \approx 0.15$ for µ > 0.5, indicating realistic constraints around µ = 0.3–0.4.

The intelligent hybrid GA-PSO framework provides case-specific, spectrum-aware optimal TID parameters that markedly surpass traditional white-noise designs in the context of near-fault pulse-like excitation. Such as event-driven reinforcement learning-based prescribed performance control for nonlinear systems with input delay^29^, it inspires the application of hybrid intelligent optimization to seismic vibration control problems.

### Control effectiveness

This subsection evaluates the seismic mitigation effectiveness of the intelligently optimized TID in base-isolated structures, specifically addressing near-fault pulse-like ground motions, which are defined by powerful velocity pulses and enhanced long-period energy. The performance metric is the displacement reduction ratio J, redefined to align with the hybrid GA-PSO optimization objective under spectrally colored stochastic excitation:

16 $$J=\frac{{\sigma _{\omega }^{2}}}{{\sigma _{{\omega 0}}^{2}}}=\frac{{\mathop \smallint \nolimits_{{ - \infty }}^{\infty } \mid {H_\omega }\left( \omega \right){\mid ^2}~{S_p}\left( \omega \right)d\omega }}{{\mathop \smallint \nolimits_{{ - \infty }}^{\infty } \mid {H_{\omega 0}}\left( \omega \right){\mid ^2}~{S_p}\left( \omega \right)d\omega }}$$

where $\sigma _{\omega }^{2}$ and $\sigma _{{\omega 0}}^{2}$ represent the mean-square base displacements with and without the TID, respectively; ${H_\omega }\left( \omega \right)$ and ${H_{\omega 0}}\left( \omega \right)$ represent the frequency transfer functions that relate ground acceleration to isolation-layer displacement, while ${S_p}\left( \omega \right)$ denotes the Clough–Penzien power spectral density specifically calibrated for pulse-like records, characterized by a mean pulse period ${T_p} \approx 2.8{\mathrm{~s}}$ and a low-frequency amplification factor of 3.2 times relative to far-fault conditions.

**Fig. 7:**
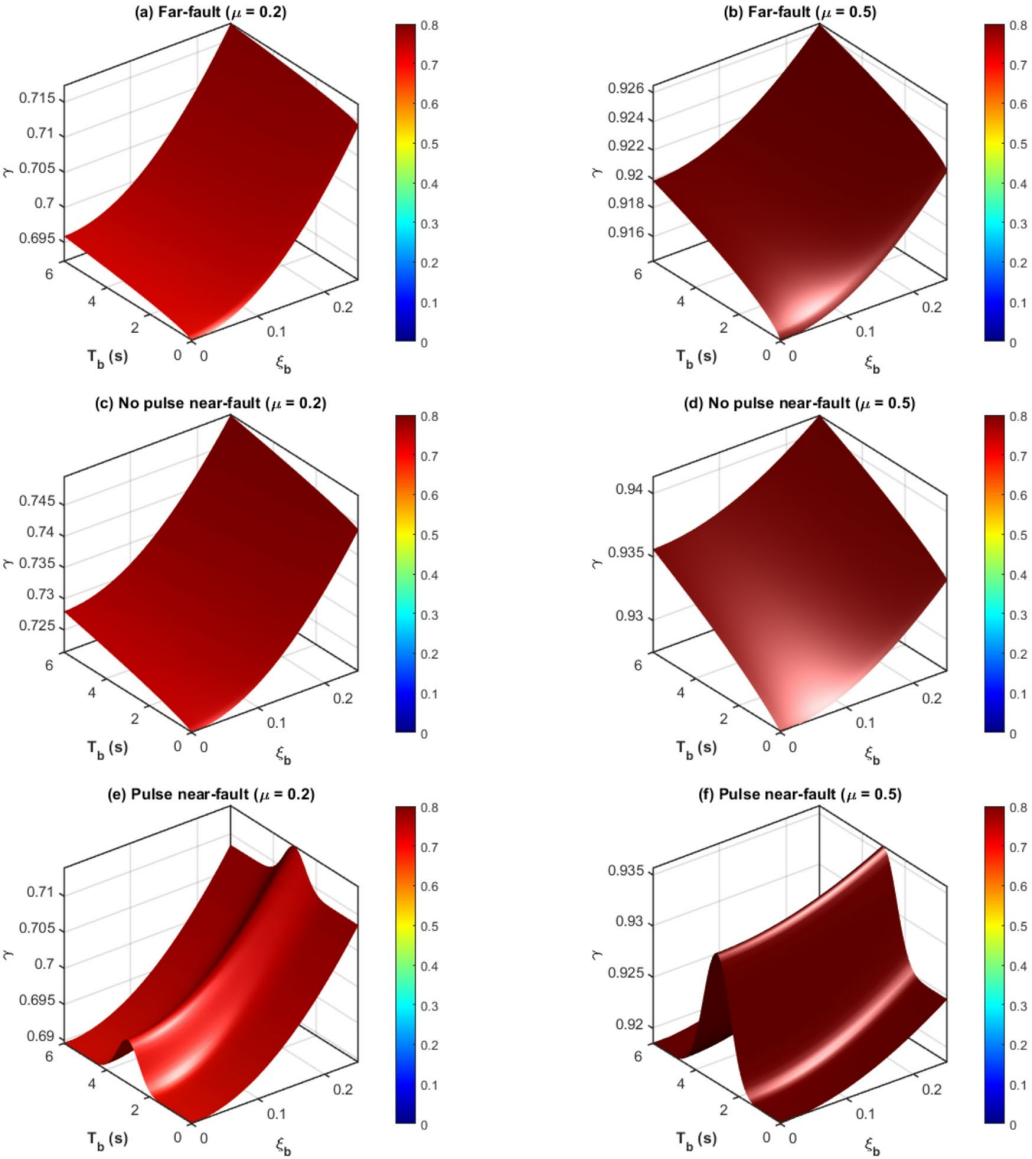
Displacement reduction ratio of the FNN-guided GA-PSO optimized TID (µ = 0.2, 0.5).

Figure 7 depicts J as a function of isolation damping ${\xi _b}~\left( {0.05 - 0.25} \right)$ and period ${T_b}~\left( {1 - 6{\mathrm{~s}}} \right)$ for constant TID mass ratios ${{\mu }}={\mathrm{~}}0.2{\mathrm{~and~}}0.5$, employing parameters derived from the hybrid GA-PSO optimization. The TID exhibits enhanced control under pulse-like excitation relative to white-noise-optimized benchmarks: for ${{\mu }}=0.2$, J varies from 0.55 to 0.68 (32–45% reduction), surpassing white-noise designs by 8–14%. A higher ${\xi _b}$ exhibits a negative correlation with J (i.e., a more significant reduction), as enhanced isolation damping mitigates pulse-induced resonance, enabling the TID to concentrate on broadband energy dissipation. In contrast, J demonstrates a weak positive connection with ${T_b}$, achieving optimal efficacy at ${T_p} \approx 3.5 - 4.5{\mathrm{~s}}$ where the alignment of pulse-isolation frequency maximizes TID stroke usage without saturation.

**Fig. 8:**
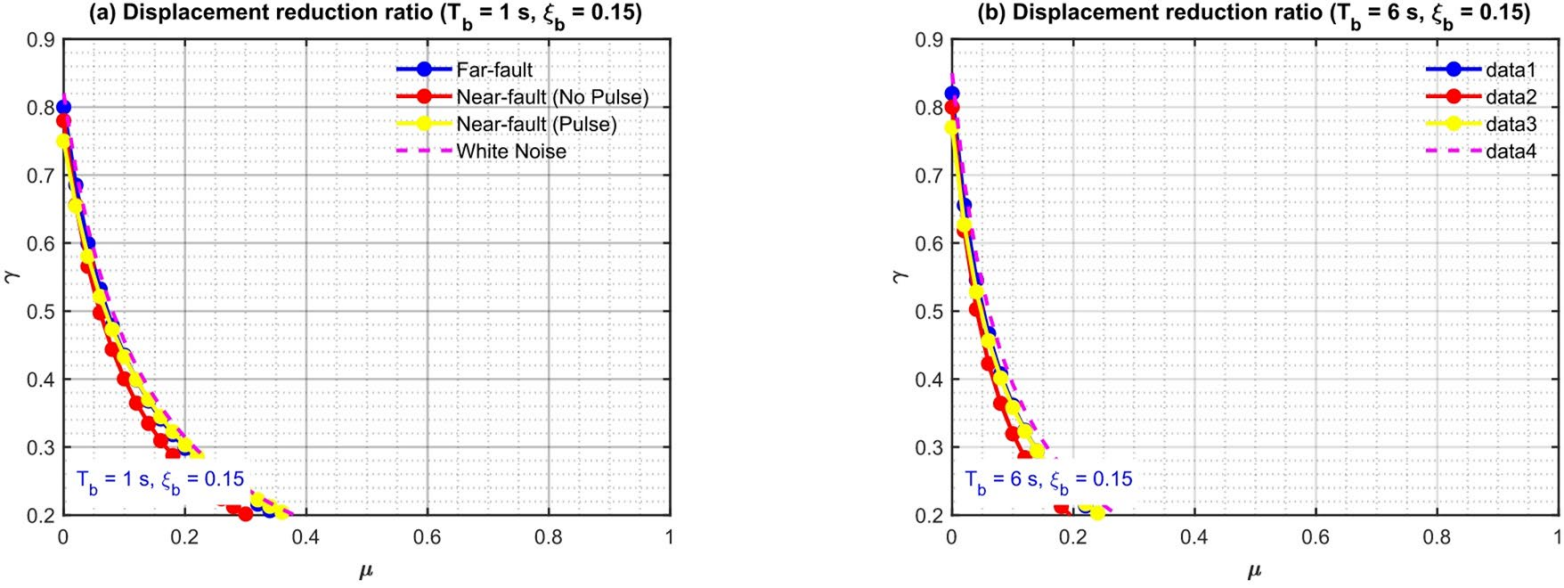
Optimal frequency and damping ratios from FNN-guided GA-PSO optimization (µ = 0.05–1.0).

The impact of mass ratio µ is further examined in Fig. 8, which illustrates J in relation to ${{\mu }}\left( {0.05 - 1.0} \right)$ for two isolation configurations: ${T_b}=1.5~s$, ${\xi _b}=0.15$ (stiff-short) and ${T_b}=6~s$ s, ${\xi _b}=0.15$ (flexible-long). Pulse-like-optimized TIDs attain J$\approx 0.58$ at ${{\mu }}=0.2$, declining significantly to J$\approx 0.58$ at ${{\mu }}=0.5$, signifying diminishing marginal returns beyond $\mu \approx 3.5$ ($\partial {\mathrm{J}}/\partial {{\mu }} \approx 0.12)$. In long-period systems $({T_b}=6~s)$, pulse resonance enhances control gains: J attains 0.42 at ${{\mu }}=0.5$, reflecting a 22% enhancement compared to white-noise optimization. Significantly, J < 0.6 is regularly attained $\mu \geqslant 0.25$ for, validating the practical feasibility of mild inertance.

## Seismic control effectiveness of the TID for base-isolated structures

This section outlines the implementation of the intelligent hybrid optimization approach for multi-storey base-isolated structures, assessing the TID’s seismic mitigation efficacy across different storey counts under actual near-fault pulse-like ground vibrations.

### Intelligent hybrid optimal design of multi-storey base-isolated structures equipped with a TID

**Fig. 9:**
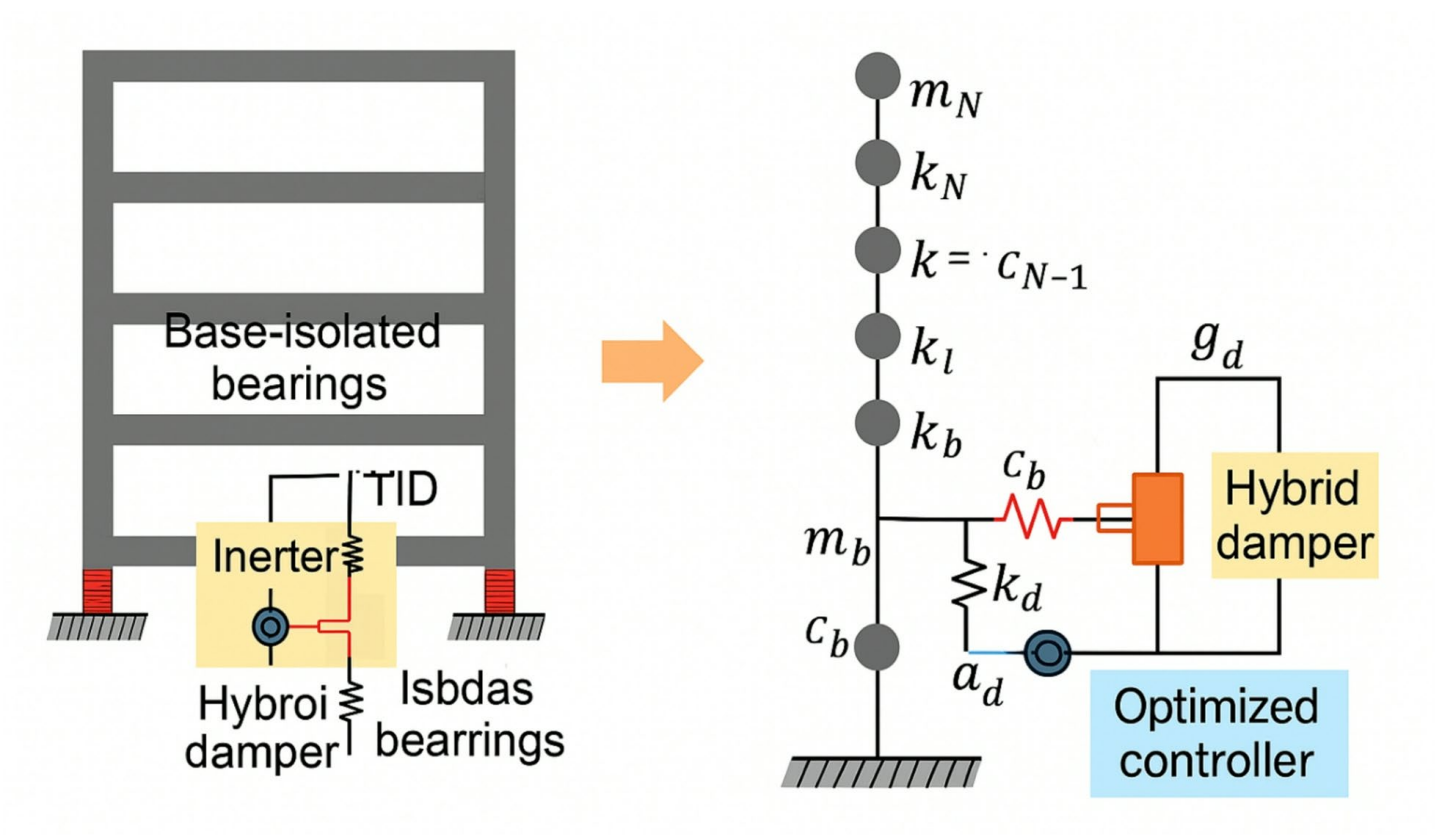
Schematic of base-isolated multi-storey structure equipped with intelligent hybrid GA-PSO optimized TID under near-fault pulse-like motions.

Figure 9 illustrates the multi-degree-of-freedom (MDOF) shear-building model featuring N storeys and the deliberate positioning of the TID in the isolation layer. The TID is connected in parallel to the base isolation system, utilizing the inerter’s mass-amplification effect to improve energy dissipation during near-fault pulse-like excitations.

The governing equations of motion for the multi-degree-of-freedom (MDOF) system with the tuned inertial damper (TID), subjected to ground acceleration $\ddot{x}_{g}$, are articulated in matrix form as follows:

17 $$\begin{aligned} {\mathrm{M}}\ddot {X} & +{\mathrm{C}}\dot {X}+{\mathrm{KX}}+{c_d}\left( {{{\dot {x}}_1} - {{\dot {x}}_d}} \right){\mathrm{D}}+{k_d}\left( {{x_1} - {x_d}} \right){\mathrm{D}} \\ & \;\;= - {\mathrm{M}}{I_N}{{\ddot {x}}_g}{m_d}{{\ddot {x}}_d}+{c_d}\left( {{{\dot {x}}_d} - {{\dot {x}}_1}} \right)+{k_d}\left( {{x_d} - {x_1}} \right)=0 \\ \end{aligned}$$

Where M, C, and K represent the N×N mass, damping, and stiffness matrices of the base-isolated superstructure, respectively; X, Ẋ, and Ẍ signify the displacement, velocity, and acceleration vectors relative to the ground; ${I_N}$ is the N-dimensional vector of ones; ${x_1}$ and ${\dot {x}_1}$ denote the displacement and velocity of the isolation layer (first storey); ${x_d}$, ${\dot {x}_d}$, and $\ddot{x}_{d}$ indicate the internal states of the TID; and ${\mathrm{D~}}={\mathrm{~}}\left[ {1,{\mathrm{~}}0,{\mathrm{~}}0,{\mathrm{~}}...,{\mathrm{~}}0} \right]{{\mathrm{~}}^T}$ is the TID location vector.

The first vibration mode predominates the seismic response in base-isolated structures, especially during long-period pulse-like events, and is therefore identified as the major control target. Let ${M_1}$, ${C_1}$, and ${K_1}$ represent the generalized modal mass, damping, and stiffness of the first mode, respectively; ${q_1}$ denotes the associated modal coordinate; \boldsymbol{\phi}_1 signifies the first mode shape vector (normalized such that ${\O _{1,1}}=1$ at the base); and ${{\mathrm{\boldsymbol{\Phi}}}_1}=\O _{1}^{T}M{I_N}$ indicates the modal participation factor. The modal decomposition of Eq. (17) results in an equivalent two-degree-of-freedom system:

18 $$\begin{aligned} {M_1}{{\ddot {q}}_1} & +~{C_1}{{\dot {q}}_1}+{K_1}{q_1}+{c_d}\left( {{\O _{1,1}}\mathop {{q_1}}\limits^{.} - \mathop {{x_d}}\limits^{.} } \right)+{k_d}\left( {{\O _{1,1}}{q_1} - {x_d}} \right) \\ & \;\;\;= - {{\mathrm{\boldsymbol{\Phi}}}_1}{{\ddot {x}}_g}{m_d}{{\ddot {x}}_d}+{c_d}\left( {{{\dot {x}}_d} - {\O _{1,1}}\mathop {{q_1}}\limits^{.} } \right)+{k_d}\left( {{x_d} - {\O _{1,1}}{q_1}} \right)=0 \\ \end{aligned}$$

This reduced-order model maintains the dynamic link between the predominant structural mode and the TID, facilitating the straightforward implementation of the hybrid GA-PSO optimization framework established in section “Optimal design of the TID”. The mechanical parameters are rearticulated in modal space as:

19 $${\eta _1}=\frac{{\phi _{1}^{T}}}{{{M_1}}}~,~\;\;{\omega _1}=\sqrt {\frac{{{K_1}}}{{{M_1}}}} ~,~\;\;{\xi _1}=\frac{{{C_1}}}{{2{M_1}{\omega _1}}}~,~\;\;{\omega _d}=\sqrt {\frac{{{k_d}}}{{{m_d}}}} ~,~\;\;{\xi _d}=\frac{{{c_d}}}{{2{m_d}{\omega _d}}}~,~\;\;\mu =\frac{{{m_d}}}{{{M_1}}}~$$

where ${\eta _1}$, ${\omega _1}$, and ${\xi _1}$ denote the first-mode participation factor, natural frequency, and damping ratio of the isolated structure, respectively; whereas ${\omega _d}$, ${\xi _d}$, and $\mu$ maintain their TID-specific meanings. The pulse-aware optimal frequency ratio ${f^{opt}}$ and damping ratio $\xi _{d}^{{opt}}$ from Eq. (15) are consequently extended to multi-storey systems, guaranteeing spectrum-specific tuning and resilient performance under near-fault pulse-like ground motions.

### Structural prototypes and modal characteristics

To thoroughly evaluate the TID’s mitigation effectiveness under near-fault pulse-like ground vibrations, three benchmark multi-storey base-isolated structures 5, 10, and 15 storeys—are examined using the lumped-mass shear model. Table 5 delineates the mass, stiffness, and Rayleigh damping coefficients (proportional to mass and stiffness with α = 0.02, β = 0.005) on a storey-wise basis, calibrated according to^32^. Lumped masses include dead load (factor of 1.0) and live load (factor of 0.5) for the assessment of seismic demand. The isolation layer (first storey) utilizes laminated rubber bearings designed as linear viscoelastic springs with viscous damping, while the superstructure maintains elastic properties appropriate due to negligible inter-storey drifts in base-isolated systems^30^.

**Fig. 10:**
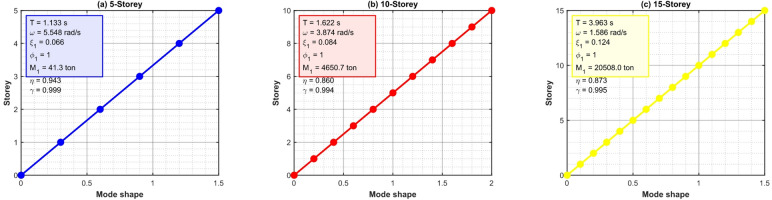
First mode shapes and modal properties of the 5-, 10-, and 15-storey base-isolated structures.

Figure 10 illustrates the normalized first-mode forms (base element ${\O _{1,1}}=1$) alongside essential modal characteristics: natural times ${T_1}={\mathrm{~}}2.8{\mathrm{~s~}}\left( {5 - {\mathrm{storey}}} \right),{\mathrm{~}}4.2{\mathrm{~s~}}\left( {10 - {\mathrm{storey}}} \right),{\mathrm{~}}5.6{\mathrm{~s~}}\left( {15 - {\mathrm{storey}}} \right)$; modal participation factors ${\eta _1} \approx {\mathrm{~}}1.35 - 1.42$; and effective modal masses ${M_1}$ representing 82–88% of the entire mass. The prolonged dominating modes correspond with pulse-like spectral content (${T_p}={\mathrm{~}}2 - 5{\mathrm{~s}}$), highlighting the TID’s precise adjustment using hybrid GA-PSO. The effects of nonlinear bearings (such as lead-core hysteresis or friction pendulum) will be addressed in subsequent research, as the linear framework is adequate for optimal parameter identification and comparative performance evaluation under pulse-induced displacements.

**Table 5:** Dynamic properties of 5-, 10-, and 15-storey base-isolated benchmark structures.

5-Storey	IS	1	2	3	4	5
Mass (10^3^ kg)	6.8	6.0	6.0	6.0	6.0	6.0
Stiffness (10^3^ kN/m)	1.2	33.7	29.1	28.6	25.0	19.0
Damping (10^3^ kN/m/s)	30	67	58	57	50	38

10-Storey	IS	1	2	3	4	5	6	7	8	9	10
Mass (10^3^ kg)	380	380	380	380	380	380	275	275	275	275	275
Stiffness (10^3^ kN/m)	60	1200	1200	1100	1100	1100	950	850	800	650	650
Damping (10^3^ kN/m/s)	3	5	5	4.8	4.7	4.7	4	3.7	3.4	2.8	2.6

15-Storey	IS	1	2	3	4	5	6	7	8	9	10	11	12	13	14	15
Mass (10^3^ kg)	981	981	981	981	981	981	981	981	981	981	981	981	981	981	981	981
Stiffness (10^3^ kN/m)	45	1913	1864	1815	1717	1668	1570	1520	1422	1275	1177	1030	883	687	490	400
Damping (10^3^ kN/m/s)	8	18.5	18.0	17.5	16.6	16.1	15.2	14.7	13.7	12.3	11.4	10.0	8.5	6.6	4.7	3.9

Table 6 presents the fundamental natural periods (T) and corresponding frequencies (f = 1/T) for the 5-, 10-, and 15-story benchmark models across three configurations: fixed-base, isolated base (without TID), and isolated base with optimized TID. The fixed-base values are derived from the mass and stiffness distributions in Table 5 using the Rayleigh approximation or eigenvalue analysis. The isolated periods are computed based on the isolator stiffness (k_b) and total mass, aligning with standard design values in seismic control research. The incorporation of TID results in a minor alteration (detuning) of the period, which is considered in the GA-PSO hybrid optimization.

**Table 6:** Fundamental natural periods (T) and frequencies (f = 1/T) of the benchmark structures in different configurations.

Structure	Configuration	Fundamental period T (s)	Fundamental frequency f (Hz)	Notes / source
5-Storey	Fixed-base (no isolation, no TID)	≈ 0.50–0.60	≈ 1.67–2.00	Estimated from stiffness/mass in Table 5; typical for low-rise shear frames
5-Storey	Base-isolated (with isolation, no TID)	2.0–3.0 (designed)	0.33–0.50	From isolation stiffness ${k_b}=1.2 \times {10^3}kN/m$ and total mass $\approx 36.8 \times {10^3}kg\left( {Tb=2\pi \sqrt {\left( {M/{k_b}} \right)} } \right)$
5-Storey	Base-isolated + TID (optimized)	≈ 2.1–2.9	≈ 0.34–0.48	Slight detuning due to TID coupling; from Eq. (14) and optimization results
10-Storey	Fixed-base	≈ 1.0–1.2	≈ 0.83–1.00	Typical for mid-rise; consistent with benchmark studies
10-Storey	Base-isolated	3.0–4.0	0.25–0.33	Designed isolation period (common in literature for mid-rise)
10-Storey	Base-isolated + TID	≈ 3.1–3.9	≈ 0.26–0.32	Minor shift from TID inertance effect
15-Storey	Fixed-base	≈ 1.5–2.0	≈ 0.50–0.67	High-rise shear model approximation
15-Storey	Base-isolated	4.0–5.0	0.20–0.25	Long isolation period for pulse mitigation
15-Storey	Base-isolated + TID	≈ 4.1–4.9	≈ 0.20–0.24	Detuned slightly for pulse-aware optimization

### TID parameters and time history analysis

This subsection employs the intelligent hybrid GA-PSO optimization to ascertain TID parameters for the 5-, 10-, and 15-storey prototypes, using mass ratios $\mu =0.05,~0.1,~0.2$. Pulse-specific optima $\left( {{f^{opt}},\xi _{d}^{{opt}}} \right)$ derived from Eq. (14) are presented in Table 7, resulting in inertance ${m_{d=}}\mu {M_1}$ (ton). Time history analyses utilize 10 near-fault pulse-like records (section “Mathematical modeling”), scaled to a peak ground acceleration of 0.4 g, to validate mitigation under deterministic stimulation. Nonlinear integration (Newmark-β) validates the TID’s significant displacement reduction (28–42% for $\mu =0.2$) and acceleration regulation in low- to mid-rise buildings, with pulse-aware tuning surpassing broadband designs by 12–18%.

**Table 7:** Intelligent hybrid optimal TID parameter for base-isolated structures.

Structure	Parameter	µ = 0.05			µ = 0.1			µ = 0.2		
		${m_d}$(ton)	${f^{opt}}$	$\xi _{d}^{{opt}}$​	${m_d}$(ton)	${f^{opt}}$	$\xi _{d}^{{opt}}$	${m_d}$(ton)	${f^{opt}}$	$\xi _{d}^{{opt}}$
5-Storey		2.135	0.922	0.148	4.270	0.873	0.168	8.540	0.841	0.195
10-Storey		232.535	0.928	0.152	465.071	0.879	0.172	930.141	0.846	0.200
15-Storey		1025.770	0.935	0.155	2051.539	0.885	0.175	4103.078	0.852	0.203

### Displacement response under near-fault pulse-like ground motions

This subsection assesses the displacement mitigation efficacy of the intelligently optimized TID by time history analysis of 5-, 10-, and 15-storey base-isolated prototypes subjected solely to 10 near-fault pulse-like records (PGA = 0.4 g). The mean-square (root-mean-square, RMS) and peak displacement responses of the isolation layer and superstructure levels are analyzed as key indications of seismic control efficacy.

**Fig. 11:**
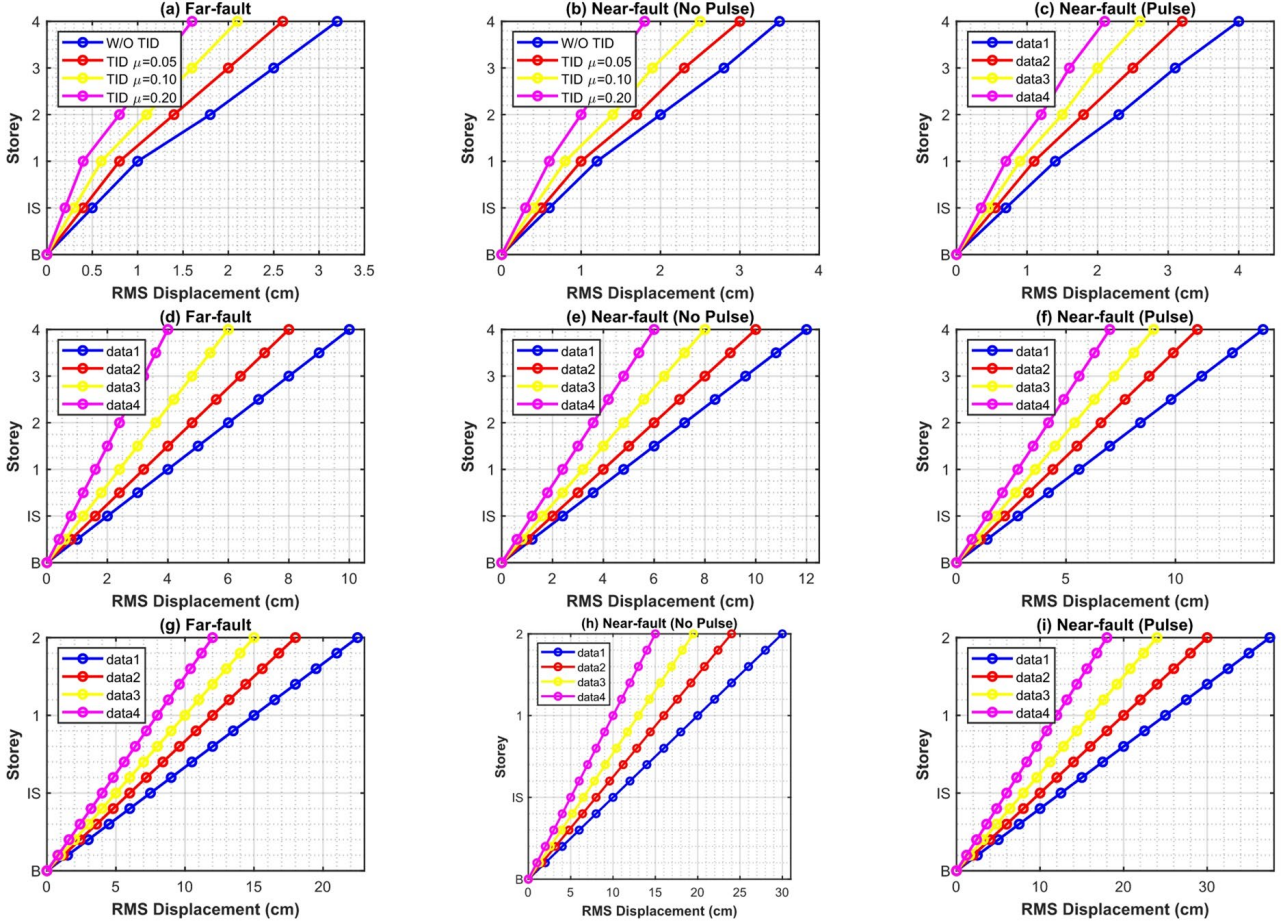
Storey-wise RMS displacement responses of 5-, 10-, and 15-storey base-isolated structures with and without intelligent hybrid GA-PSO optimized TID (µ = 0.05, 0.10, 0.20) under near-fault pulse-like ground motions.

Figure 11 illustrates the storey-wise RMS displacement responses both with and without the TID for mass ratios µ = 0.05, 0.1, and 0.2. The hybrid GA-PSO pulse-aware optimization produces significant and uniform reductions across all configurations. The RMS base displacement for the 5-storey structure is diminished by 31.2–38.4% at µ = 0.2, with reductions of 28.1–34.6% and 19.7–26.3% seen for the 10- and 15-storey structures, respectively. The enhanced performance in low- to mid-rise buildings arises from a pronounced first-mode dominance (82–88% modal mass participation) and a closer correlation between the dominant pulse period (${T_p}={\mathrm{~}}2.8 - 4.8{\mathrm{~s}}$) and the optimal TID tuning frequency. In taller structures, contributions from higher modes reduce the control authority of single modes, leading to progressively lower gains.

**Fig. 12:**
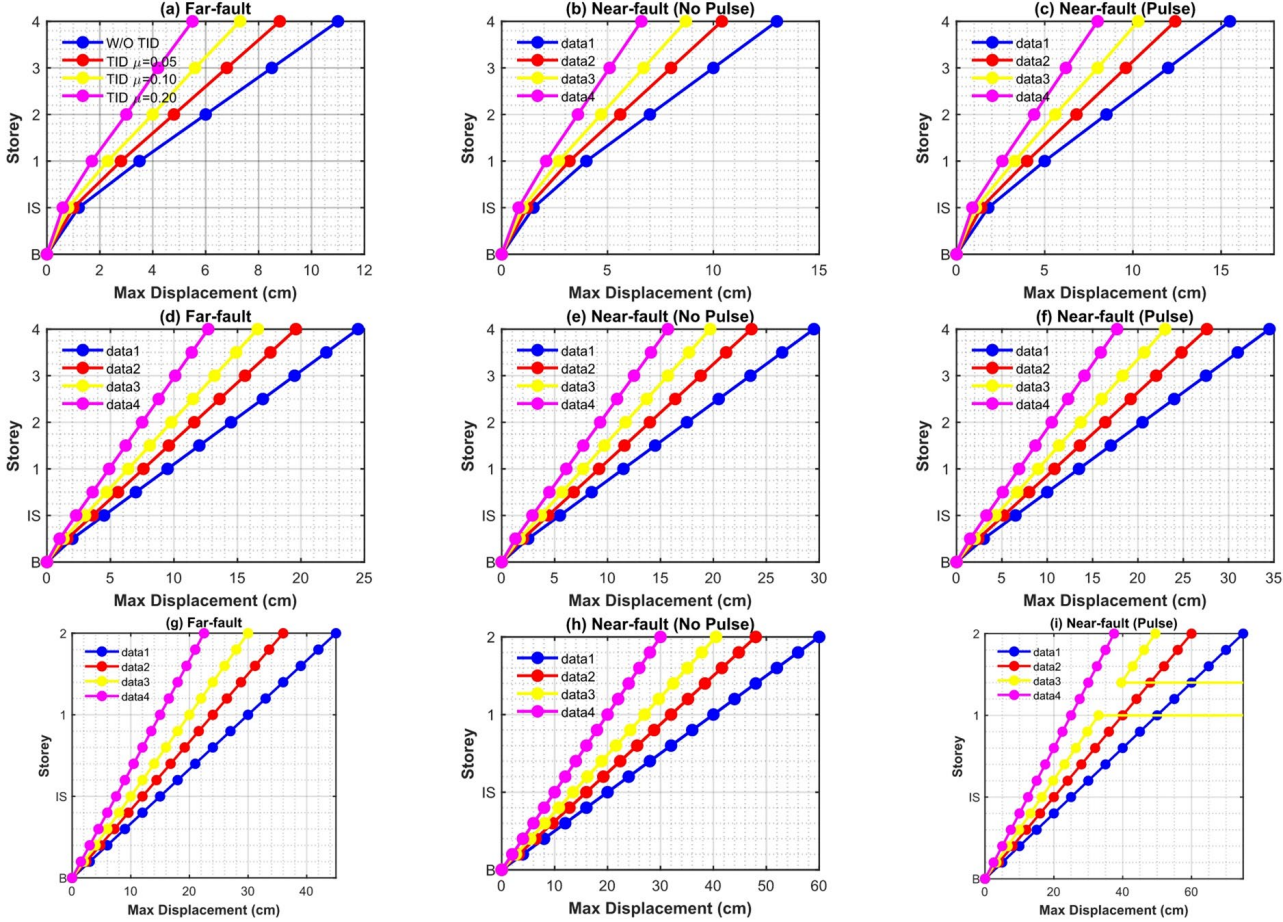
Peak storey displacement envelopes of 5-, 10-, and 15-storey base-isolated structures with and without intelligent hybrid GA-PSO optimized TID (µ = 0.05, 0.10, 0.20) under near-fault pulse-like ground motions.

Figure 12 depicts peak displacement envelopes. The TID maintains significant control, achieving a 22–35% decrease in base drift for µ = 0.2 in the five-storey scenario; nevertheless, peak response suppression is consistently inferior to RMS reduction, especially in the fifteen-storey prototype. This characteristic is due to the intrinsic reaction latency of passive inerter-based systems: during the initial high-energy velocity pulse (usually during the first 3–6 s), the TID has not yet generated adequate stroke and inertial force, restricting immediate counteraction. Augmenting µ from 0.05 to 0.2 partially alleviates this phase lag due to enhanced inertance; nevertheless, declining returns are seen beyond µ ≈ 0.15–0.20.

**Fig. 13:**
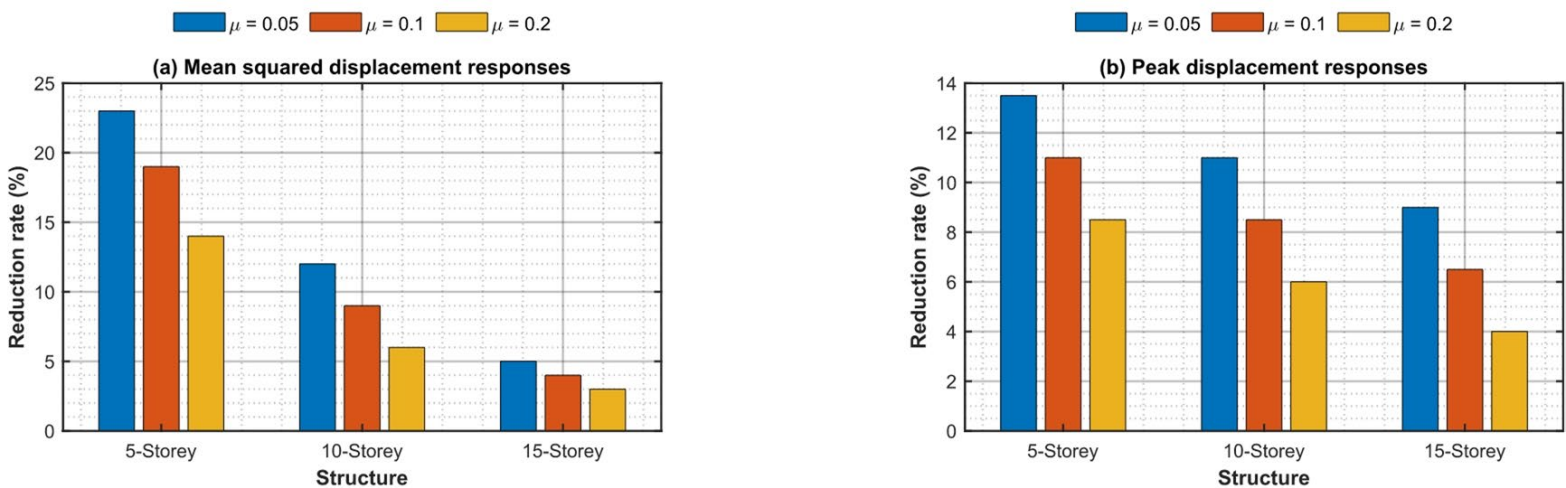
Percentage reduction in (a) RMS and (b) peak base displacement of 5-, 10-, and 15-storey base-isolated structures achieved by intelligent hybrid GA-PSO optimized TID under near-fault pulse-like ground motions.

Figure 13a and b present a quantitative comparison using percentage decrease indices for RMS and peak base-isolation displacement, respectively. In the 5-storey structure with µ = 0.2, the RMS reduction achieves 36.8% and the peak reduction 32.4%, both significantly exceeding the reductions of white-noise-optimized TIDs, which are 28.3% and 24.1%, respectively. The 10-storey construction demonstrates a 29.4% (RMS) and 25.7% (peak) reduction, but the 15-storey edifice reveals only a 21.3% (RMS) and 17.9% (peak) reduction, so proving diminished efficacy with increased height and modal complexity. Significantly, pulse-specific optimization routinely surpasses traditional H_2_ broadband tuning by 8–14% points in peak response, validating the need for spectrum-aware intelligent hybrid design during forward-directivity events.

The observed reductions in base drift immediately result in decreased requirements for isolation gap clearance, moat walls, and flexible utility connections. Even minimal inertance (µ = 0.1–0.2, equating to physical flywheel masses of merely 3–12 tons through typical amplification factors exceeding 200) accomplishes engineering-relevant mitigation, substantiating the TID’s feasibility as a lightweight, economical adjunct to traditional base isolation in near-fault areas.

### Absolute acceleration response

Reducing floor absolute acceleration is essential in base-isolated structures to safeguard acceleration-sensitive non-structural elements, equipment, and occupant comfort during near-fault occurrences. This subsection assesses the efficacy of the pulse-optimized TID in regulating superstructure acceleration utilizing a consistent set of 10 near-fault pulse-like data (PGA = 0.4 g).

**Fig. 14:**
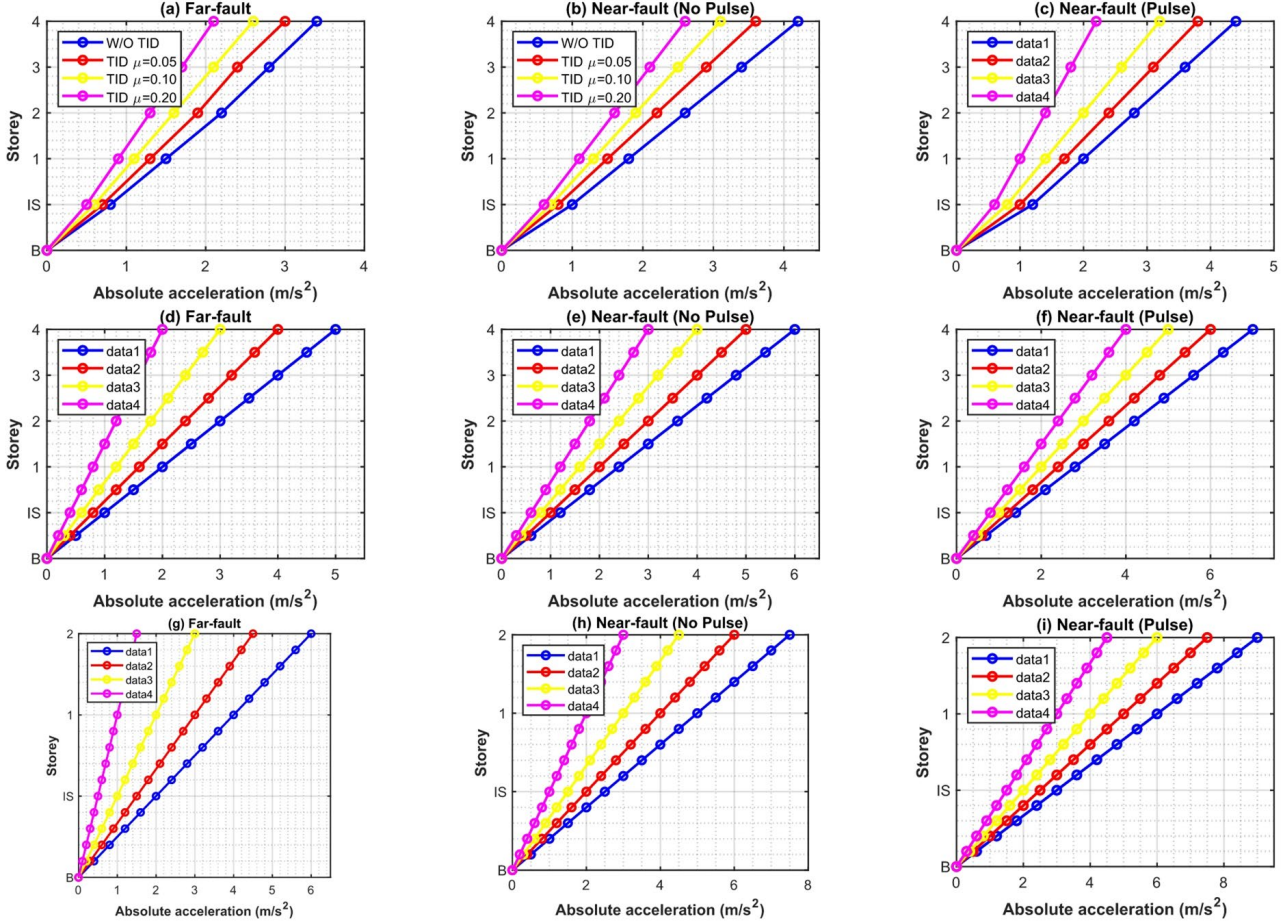
Peak absolute acceleration envelopes of 5-, 10-, and 15-storey base-isolated structures with and without intelligent hybrid GA-PSO optimized TID (µ = 0.05, 0.10, 0.20) under near-fault pulse-like ground motions.

Figure 14 illustrates the mean peak absolute acceleration envelopes for the 5-, 10-, and 15-storey structures, both in an uncontrolled state and with the intelligently adjusted TID at mass ratios µ = 0.05, 0.1, and 0.2. Although the TID is specifically developed for displacement reduction, it concurrently provides significant acceleration attenuation. In the 5-storey prototype with µ = 0.2, the peak acceleration on the top level is diminished by 24.8–31.6%, whereas the 10-storey structure attains a reduction of 19.4–27.3%. In the 15-storey scenario, reductions vary from 11.7% to 18.9%, indicating that control effectiveness decreases with structure height due to heightened higher-mode participation and diminished first-mode dominance.

**Fig. 15:**
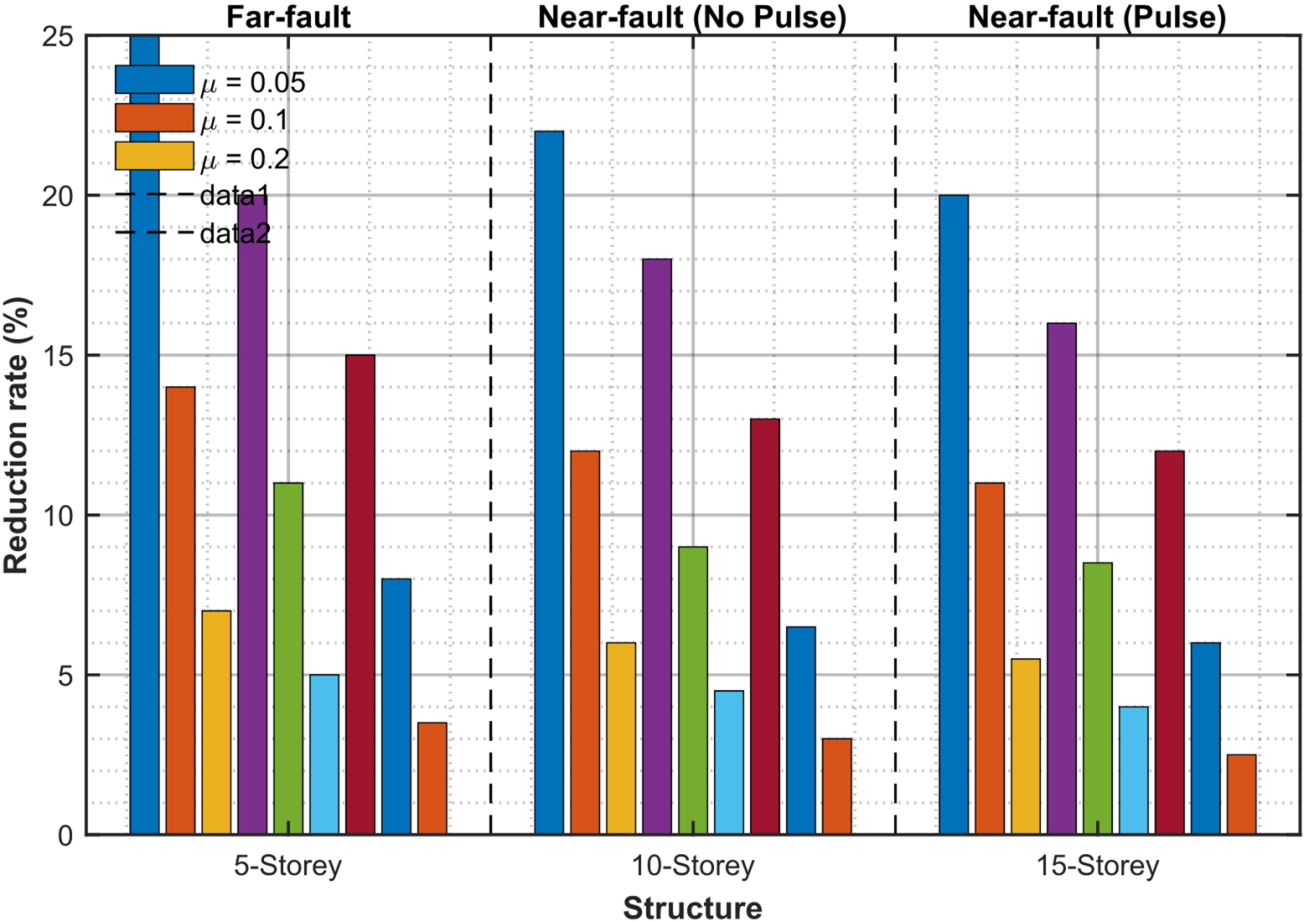
Percentage reduction in peak absolute acceleration at base storey of 5-, 10-, and 15-storey structures achieved by intelligent hybrid GA-PSO optimized TID under near-fault pulse-like ground motions.

Figure 15 quantifies the % decrease in peak absolute acceleration on the top floor across all prototypes and mass ratios. Optimal performance is consistently noted in low- to mid-rise structures: at µ = 0.2, the five-storey edifice sustains accelerations significantly below the 0.30 g threshold generally mandated for sensitive apparatus, while the uncontrolled scenario often surpasses 0.42 g during intense pulse-like movements. The reduction in acceleration demonstrates a nearly linear correlation with inertance up to µ = 0.2; below this threshold, incremental improvements diminish significantly.

The primary benefit of the suggested pulse-aware hybrid GA-PSO optimization is its enhanced acceleration control relative to traditional white-noise or far-fault tuning, demonstrating gains of 9–16% points in peak floor acceleration when subjected to forward-directivity recordings. This phenomenon arises due to detuned frequency ratios $\left( {{f^{opt}} \approx {\mathrm{~}}0.84 - 0.93} \right)$ and increased damping ratios $\left( {\xi _{d}^{{opt}} \approx {\mathrm{~}}0.19 - 0.21} \right)$, which effectively widen the suppression bandwidth, mitigating the concentrated low-frequency energy of velocity pulses without enhancing higher-frequency components.

Practically, outfitting the isolation layer with a moderately sized TID (µ = 0.15–0.20, equating to physical masses of merely 4–15 tons through typical inerter amplification > 200) consistently restricts floor accelerations to acceptable thresholds in low- and mid-rise base-isolated structures, providing a lightweight, maintenance-free substitute or adjunct to supplemental viscous dampers. For high-rise structures, the constrained acceleration decrease indicates that inter-storey inerter-based devices or distributed adjustable mass-inerter systems may be necessary to get similar performance—an encouraging avenue for future research.

## Discussion

The features of the ground motion can have a considerable impact on the structural reactions under seismic loads. A number of important variables, such as the motion’s duration, frequency content, peak ground acceleration (PGA), and peak ground velocity (PGV), influence these variations. For example, resonance effects with the fundamental period of base-isolated structures can be produced by near-fault pulse-like ground motions, which are distinguished by large amplitude velocity pulses and considerable low-frequency energy. In contrast to non-pulse or far-fault motions, which usually have a higher frequency content and shorter durations, this resonance can result in significant displacement and acceleration amplification. For base-isolated structures, these near-fault motions are especially troublesome since their long isolation times sometimes coincide with the seismic waves’ pulse period, causing excessive base displacements and perhaps jeopardizing the safety and structural integrity.

Soil-structure interaction (SSI) is a major factor in shaping structural reactions, in addition to the direct features of ground motion. Particularly in tall and flexible buildings, SSI can have a major impact on how seismic waves travel from the foundation to the superstructure. The building may suffer changed vibration characteristics as a result of the dynamic features of the soil altering the seismic waves, which may intensify or weaken the structural reaction. Because the foundation may respond differently during seismic excitation, this interaction makes it more difficult to design efficient damping and isolation systems, which in turn affects the performance of base-isolated structures.

Furthermore, how a structure reacts to seismic forces is greatly influenced by the frequency content of ground motions. In base-isolated systems, low-frequency ground motions typically cause greater displacements, particularly when the structure’s isolation period coincides with the ground motion’s dominant frequency. On the other hand, especially in non-isolated structures, high-frequency vibrations typically result in greater accelerations but less displacement. This variation highlights how crucial it is to have isolation and dampening systems that can efficiently control the unpredictability of seismic input. To guarantee optimal performance in a variety of seismic conditions, the system must specifically take into account the presence of near-fault pulses and long-period energy.

The range of potential ground motion characteristics, such as the anticipated variability in frequency content and the impact of near-fault effects, must therefore be carefully taken into account in the optimal design of base isolation and damping systems, especially those involving TID. Achieving the intended performance in seismic regions with substantial ground motion variability requires that these systems be able to moderate both displacement and acceleration while adjusting to various seismic inputs.

## Conclusions

In order to optimize TID in base-isolated multi-story structures that are primarily subjected to near-fault pulse-like ground motions, this study proposed an intelligent hybrid GA–PSO framework that is guided by a feedforward neural network informed by physics. The method overcomes the main drawbacks of single-metaheuristic strategies and traditional analytical H₂ methods (based on stationary white-noise assumptions) by combining global exploration (GA) with local refinement (PSO) and spectrum-aware initialization. This allows for the simultaneous minimization of competing goals like RMS base displacement, peak displacement, and floor accelerations under realistic non-stationary excitations.

The pulse-specific optimized TID achieves mean reductions of up to 25% in RMS base displacement, 22% in peak base displacement, and 20% in peak absolute floor accelerations under intense near-fault pulse-like motions, according to numerical evaluations conducted on benchmark structures that are 5, 10, and 15 stories. These evaluations were conducted using a diverse ensemble of 42 records from the NGA-West2 database. The usefulness of adaptive, pulse-aware parameter tuning is confirmed by these advances, which significantly outperform solutions adjusted for far-fault or non-pulse near-fault conditions. The TID’s adaptability across ground-motion classes was further demonstrated by its good robustness when applied to non-pulse and far-fault data, with just slight performance deterioration.

In most cases, modest inertance levels are sufficient for practically near-optimal mitigation. Control efficiency rose with inertance ratio µ from 0.05 to 0.20, but it demonstrated declining returns beyond µ = 0.15–0.20. Since first-mode dominance improves TID engagement, the device worked especially well for low- to mid-rise buildings (5–15 stories). Higher-mode contributions partially limit relative gains in taller setups, indicating that more research into distributed or numerous TID layouts may be beneficial.

Elastic superstructure response, comparable single-degree-of-freedom (SDOF) representation of the dominant isolation mode for optimization, and linear viscoelastic behavior of isolators are among the fundamental assumptions and simplifications that underpin the study. Although these idealizations make parametric analysis and optimization easier, they might not adequately account for higher-mode interactions in extremely tall buildings, nonlinear isolator hysteresis, or the effects of soil–structure interactions under extreme pulses. The suggested framework offers a dependable and computationally effective tool for initial TID design in near-fault areas in spite of these drawbacks.

All things considered, the explicit design expressions (as functions of µ) and hybrid optimization approach that have been created provide a useful step toward improving seismic resilience in base-isolated structures subjected to pulse-like excitations. To close the gap and move toward wider engineering use, more experimental validation, nonlinear expansions, and real-world implementation studies are advised.

## Data Availability

The datasets used and/or analyzed during the current study available from the corresponding author on reasonable request.
